# Structure based biophysical characterization of the PROPPIN Atg18 shows Atg18 oligomerization upon membrane binding

**DOI:** 10.1038/s41598-017-14337-5

**Published:** 2017-10-25

**Authors:** Andreea Scacioc, Carla Schmidt, Tommy Hofmann, Henning Urlaub, Karin Kühnel, Ángel Pérez-Lara

**Affiliations:** 1 Research Group Autophagy, Max-Planck-Institute for Biophysical Chemistry, Am Faßberg 11, 37077 Göttingen, Germany; 2 Interdisciplinary research center HALOmem, Martin Luther University Halle-Wittenberg, Kurt-Mothes-Str. 3, 06120 Halle, Germany; 3 Bioanalytical Mass Spectrometry Group, Max-Planck-Institute for Biophysical Chemistry, Am Faßberg 11, 37077 Göttingen, Germany; 4 Bioanalytics Group, University Medical Center Göttingen, Robert-Koch-Strasse 40, 37075 Göttingen, Germany; 5 Department of Neurobiology, Max-Planck-Institute for Biophysical Chemistry, Am Faßberg 11, 37077 Göttingen, Germany; 6 Nature Communications, 4 Crinan Street, London, N1 9XW United Kingdom

## Abstract

PROPPINs (β-propellers that bind polyphosphoinositides) are PtdIns3P and PtdIns(3,5)P_2_ binding autophagy related proteins. They contain two phosphatidylinositolphosphate (PIP) binding sites and a conserved FRRG motif is essential for PIP binding. Here we present the 2.0 Å resolution crystal structure of the PROPPIN Atg18 from *Pichia angusta*. We designed cysteine mutants for labelling with the fluorescence dyes to probe the distances of the mutants to the membrane. These measurements support a model for PROPPIN-membrane binding, where the PROPPIN sits in a perpendicular or slightly tilted orientation on the membrane. Stopped-flow measurements suggest that initial PROPPIN-membrane binding is driven by non-specific PIP interactions. The FRRG motif then retains the protein in the membrane by binding two PIP molecules as evident by a lower dissociation rate for Atg18 in comparison with its PIP binding deficient FTTG mutant. We demonstrate that the amine-specific cross-linker Bis(sulfosuccinimidyl)suberate (BS3), which is used for protein-protein cross-linking can also be applied for cross-linking proteins and phosphatidylethanolamine (PE). Cross-linking experiments with liposome bound Atg18 yielded several PE cross-linked peptides. We also observed intermolecular cross-linked peptides, which indicated Atg18 oligomerization. FRET-based stopped-flow measurements revealed that Atg18 rapidly oligomerizes upon membrane binding while it is mainly monomeric in solution.

## Introduction

During macroautophagy, denoted here as autophagy, an expanding isolation membrane also called the phagophore engulfs cytoplasmic content. After fusion of the isolation membrane a double-membraned vesicle, the autophagosome is formed, which then fuses with the vacuole where its content is degraded. Autophagy is important for maintaining cellular homeostasis and is upregulated during starvation^1^. Various non-selective and selective autophagy subtypes exist. Atg18 is a core autophagy protein that belongs to the PROPPIN family (β-propellers that bind polyphosphoinositides)^2^. WIPI1 (WD-40 repeat containing protein that interacts with PtdIns)^3^ and WIPI2 are the mammalian Atg18 orthologues^4^. Yeast contains two additional PROPPINs: Atg21 and Hsv2 (homologous with swollen vacuole phenotype 2). The three yeast PROPPINs are homologous but differ in their autophagic subtype specificities^5–8^.

PROPPINs specifically bind PtdIns3P and PtdIns(3,5)P_2_. Early mutagenesis studies showed that a conserved FRRG motif is essential for PIP binding of PROPPINs^7,9–12^. The Hsv2 crystal structure revealed that PROPPINs contain two PIP binding sites and fold as seven bladed β-propellers^13–15^. The two PIP binding sites are localized on the rim of the β-propeller and each FRRG arginine is part of one PIP binding site. The membrane insertion loop 6CD protrudes from the β-propeller core and is required for membrane binding in addition to the two PIP binding sites^14,16^. Phosphorylation of the membrane insertion loop modulates membrane binding of Atg18^17^.

PROPPIN membrane binding is also membrane curvature dependent. We previously showed that Hsv2 binds with an approx. 20 times higher affinity to small unilamellar vesicles (SUVs) containing either PtdIns3P or PtdIns(3,5)P_2_ than large unilamellar vesicles (LUVs), which had diameters of approx. 40 nm and 100 nm, respectively^16^. PROPPINs were proposed to bind perpendicular to the membrane through their two PIP binding sites on the rim and the membrane insertion loop 6CD^13–16^.

PIP binding is essential for *in vivo* PROPPIN function. Atg18 localizes in a PtdIns3P dependent manner to the preautophagosomal structure (PAS), the site where autophagosome formation is initiated^10,18^. Atg18 interacts with Atg2 at the PAS and complex formation is required for autophagosome formation^19,20^. The mammalian Atg18 orthologue WIPI2b binds Atg16L1 and recruits the Atg12-Atg5-Atg16L1 complex during autophagosomal membrane biogenesis^21^. The three yeast PROPPINs also bind PtdIns3P-dependent to endosomes, however their functions there are still unknown^22^. PtdIns(3,5)P_2_ binding of Atg18 mediates its localization to the vacuole^9^. Atg18 has a regulatory role as part of the Fab1-containing PtdIns3P 5-kinase complex at the vacuole^23,24^. Yeast *atg18*Δ cells have enlarged vacuoles^9,23^ and mutagenesis studies showed that both Atg18 PIP binding sites are important for maintaining vacuolar homeostasis^13^.

We set out to gain further insights into the mechanism of PROPPIN-membrane binding. Here we worked with recombinant expressed *Pichia angusta* (*Hansenula polymorpha*) Atg18 because *S. cerevisiae* Atg18 is not soluble when expressed in *E. coli* ^25^. *Pichia angusta* (Pa) and *Pichia pastoris* (Pp) are methylotrophic yeasts. PaAtg18 and PpAtg18 share 60% sequence identity. PpAtg18 was previously characterised *in vivo* and is required for autophagy and pexophagy^6^. We crystallised PaAtg18 and determined its structure at 2.0 Å resolution. Based on this structure we designed mutants to probe the distances of selected Atg18 residues to the membrane to experimentally test the proposed model for PROPPIN-membrane binding. We used stopped-flow measurements to study the influence of the FRRG motif on the kinetics of PROPPIN-membrane binding. Furthermore, we performed liposome-Atg18 cross-linking experiments and detected several peptide-lipid cross-links by mass spectrometry. We also identified several intermolecular cross-linked Atg18 peptides indicating Atg18-Atg18 interactions. Native mass spectrometry confirmed the formation of Atg18 oligomers. We further investigated these findings employing FRET based stopped-flow measurements, which showed that Atg18 rapidly oligomerizes upon membrane binding.

## Results

### Pichia angusta Atg18 structure

**Figure 1:**
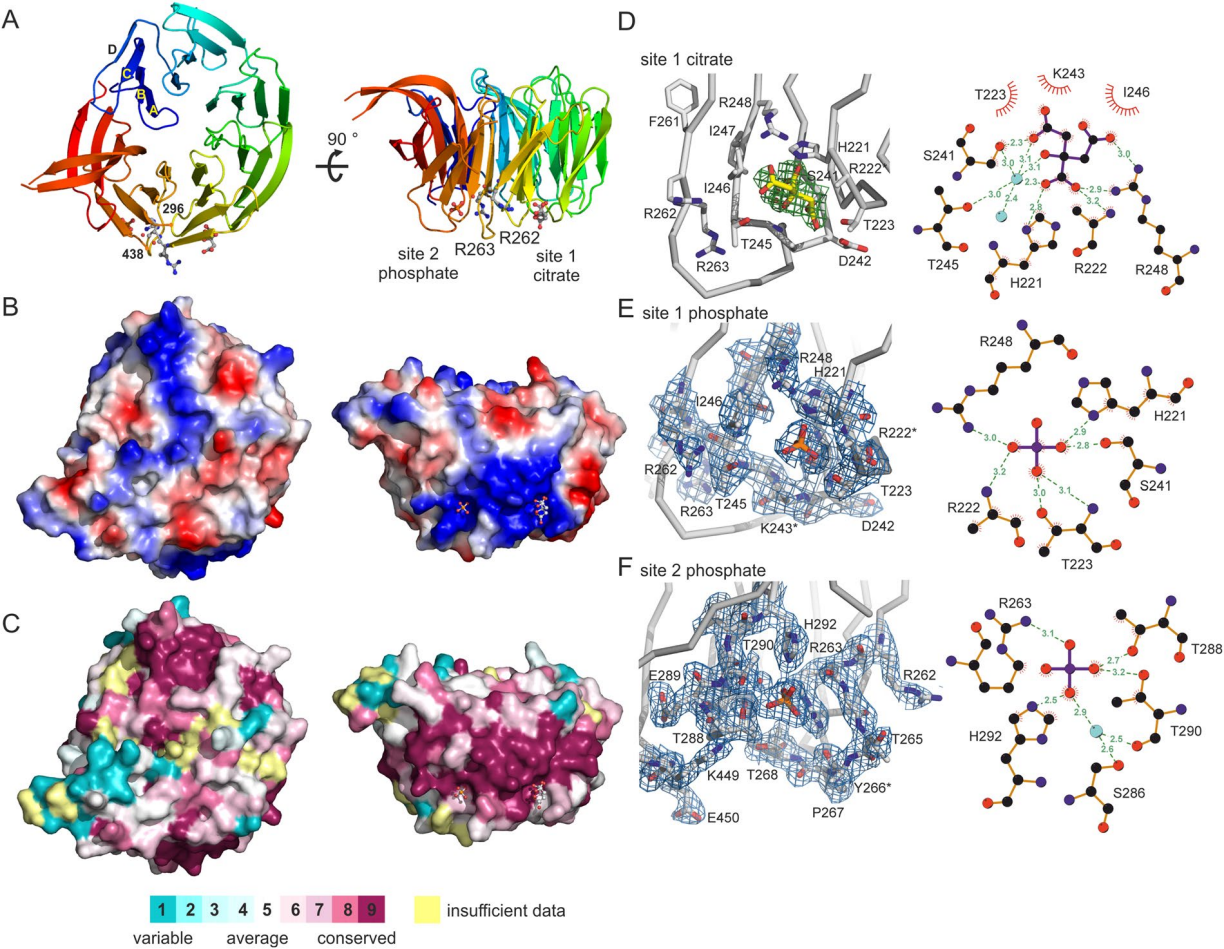
*Pichia angusta* Atg18 structure. (**A**) Cartoon representations of Atg18 rainbow-colored from blue at the N-terminus to red at the C-terminus. The ligands and FRRG arginine side chains are shown as ball and sticks. Loop 6CD (297–437) is missing and marked through the last ordered residues 296 and 438. Panels (**A**–**C**) show the protein in the same orientation. Surface representations of Atg18 showing (**B**) surface charges with blue depicting positive and red negative charges and (**C**) conservation of the molecule. Conservation analysis was done with Consurf^46^. Proteins used for the sequence alignment are listed in the Methods section. (**D**) Close up on site 1 with bound citrate. The omit mFo-DFc difference map, where m is the figure of merit and D the sigma A weighting factor has a contour level of σ = +2.5. Scheme showing ligand protein interactions was adapted from LigPlot^+^. Hydrogen bonds and salt bridges between ligand and protein are shown with dashed lines and distances are given in Ångstrom. Water molecules are depicted as cyan spheres. (**E**) Site 1 with a bound phosphate ion. The 2.5 Å resolution 2mFo-DFc map is contoured at 1.0 σ. (**F**) PIP binding site 2 with a bound phosphate ion. The 2.0 Å resolution 2mFo-DFc electron density map is contoured at 1.0 σ. Residues with disordered side chains are marked with an asterisk.

Well-diffracting *Pichia angusta* Atg18 crystals (denoted as Atg18 hereafter) were obtained through *in situ* proteolysis crystallization with proteinase K. The 2.0 Å resolution structure was determined by molecular replacement and contains two molecules in the P1 unit cell (Table 1, Supplementary Figure 2). Atg18 folds as a seven bladed β-propeller with a non-velcro propeller closure topology as previously observed for the Hsv2 homologs^13–15^ (Fig. 1A). The Atg18 and *Kluyveromyces lactis (K. lactis)* Hsv2 structures (PDB accession code: 4AV9) superimpose with an r.m.s.d. of 1.5 Å and share 24.8% sequence identity (Supplementary Figure 1). The innermost β-strands of the blades are denoted as A and the outer strands as D. Strands 7A and 7B are larger than the other strands of the β-propeller and protrude, however this area is not well conserved among the Atg18 orthologues (Fig. 1A,D). The membrane insertion loop 6CD comprising residues 297–437 is missing from the model (Fig. 1A).

**Table 1:** Data collection and refinement statistics.

Space group	citrate bound Atg18	phosphate bound Atg18
	P1	P1
*cell dimensions*		
a, b, c (Å)	57.8, 58.0, 62.2	58.2, 58.3, 62.3
α, β, γ (°)	84.2, 81.2, 87.0	83.7, 80.9, 86.8
resolution (highest res. shell) (Å)	41.3–2.0 (2.1–2.0)	45.5–2.5 (2.7–2.5)
R_factor_ (%)	5.7 (36.1)	13.3 (64.5)
wavelength (Å)	1.000	0.979
no. of observed reflections/unique reflections	102367/51797	200927/27376
I/σ (I)	9.6 (2.2)	12.8 (3.4)
completeness (%)	94.5 (86.3)	98.3 (96.5)
Wilson B factor (Å^2^)	32.1	41.2
**Refinement**		
molecules per unit cell	2	2
R_work_/R_free_ (%)	21.4/26.9	22.1/26.5
residues included in model	A: 32–179, 201–296, 438–467, 485–524	A:33–179, 201–297, 438–467, 484–424
	B: 32–180, 201–297, 438–467, 484–525	B: 33–179, 201–297, 438–467, 485–525
number of protein atoms	4835	4751
number of ligand atoms	36	24
number of water molecules	385	119
*B-factors (Å* ^2^) overall	29.0	39.4
protein	28.5	39.4
ligand	42.3	49.2
water	33.7	35.0
*r.m.s.d*.		
bond lengths (Å)	0.010	0.003
bond angles (°)	1.20	0.65
Ramachandran favored/allowed/outliers (%)	97.4/2.4/0.2	96.6/3.4/0

The conserved FRRG motif is localized on strand 5D and the loop connecting it with strand 6A. The FRRG F261 on strand 5D forms an off-centre parallel stacking interaction with F294 on strand 6C stabilizes the β-propeller fold and is not involved in membrane binding. The glycine in the motif is a requirement for steric reasons. The two FRRG arginines point into different directions, R262 is oriented towards PIP binding site 1 and R263 is part of PIP binding site 2 (Fig. 1A). Site 1 contains a citrate molecule and site 2 binds a phosphate ion both originating from the crystallization buffer.

When Atg18 crystals were transferred to a 0.4 M sodium dihydrogen phosphate containing cryoprotectant lacking citrate the citrate ion bound in site 1 was replaced by a phosphate. The structure with phosphate ions bound to both PIP binding sites was determined at 2.5 Å resolution (Table 1, Fig. 1E). In both phosphate and citrate site 1 structures salt bridges are formed between the conserved residues R248 and H221 and either the phosphate or carboxylate groups, respectively (Fig. 1D,E). Furthermore, both ligands form hydrogen bonds with S241 and the amide nitrogen of R222. When comparing the positions of the two ligands towards each other, the phosphate ion sits between the middle carboxylate group of the citrate and the carboxylate forming a hydrogen bond with S241.

The phosphate ion bound in site 2 extensively interacts with the protein. It forms salt bridges with H292 and FRRG R263 and hydrogen bonds with T288 and T290 (Fig. 1F). The two PIP binding sites are positively charged and part of a highly conserved region on the rim of the propeller comprising blades four, five and six (Fig. 1B, C). The region including loops 2AB and 2CD, which are localized opposite of the two PIP binding sites, is also highly conserved among the Atg18 orthologues pointing towards a functional role for this region, for example in protein-protein interactions (Fig. 1C, left panel). Indeed, mutagenesis studies with *S. cerevisiae* Atg18 showed that loop regions 2AB and 2BC are important for Atg2-Atg18 complex formation^15^.

### Comparison of Atg18 and Atg18 FTTG membrane binding kinetics

The conserved FRRG motif is essential for membrane binding of PROPPINs^7,9,10^. A mutation of the FRRG motif to FTTG abolishes PIP binding of PROPPINs^9–11,19^. We analyzed the kinetics of Atg18 and Atg18 FTTG membrane binding with stopped-flow measurements. The single cysteine mutants S448C and S448C with FTTG were used for site-directed labelling with the FRET donor dye Alexa Fluor 488. S448 is located in the loop connecting strands 6D and 7A near PIP binding site 2.

Measurements were done with large unilamellar vesicles (LUVs) containing 1% PtdIns(3,5)P_2_, which were labelled with the FRET acceptor dye Texas Red. These LUVs consisted of dioleoylphosphatidylcholine (DOPC)/dioleoylphosphatidylethanolamine (DOPE)/Texas Red-PE/PtdIns(3,5)P_2_ (79:18:2:1, molar ratio) and had diameters in the range of 120–140 nm (Supplementary Figure 3). The observed fluorescence increase is only due to FRET between the Alexa Fluor 488 and the labelled vesicles because no light scattering was observed at this protein concentration and the highest LUV concentration used in these measurements (Supplementary Figure 4).

**Figure 2:**
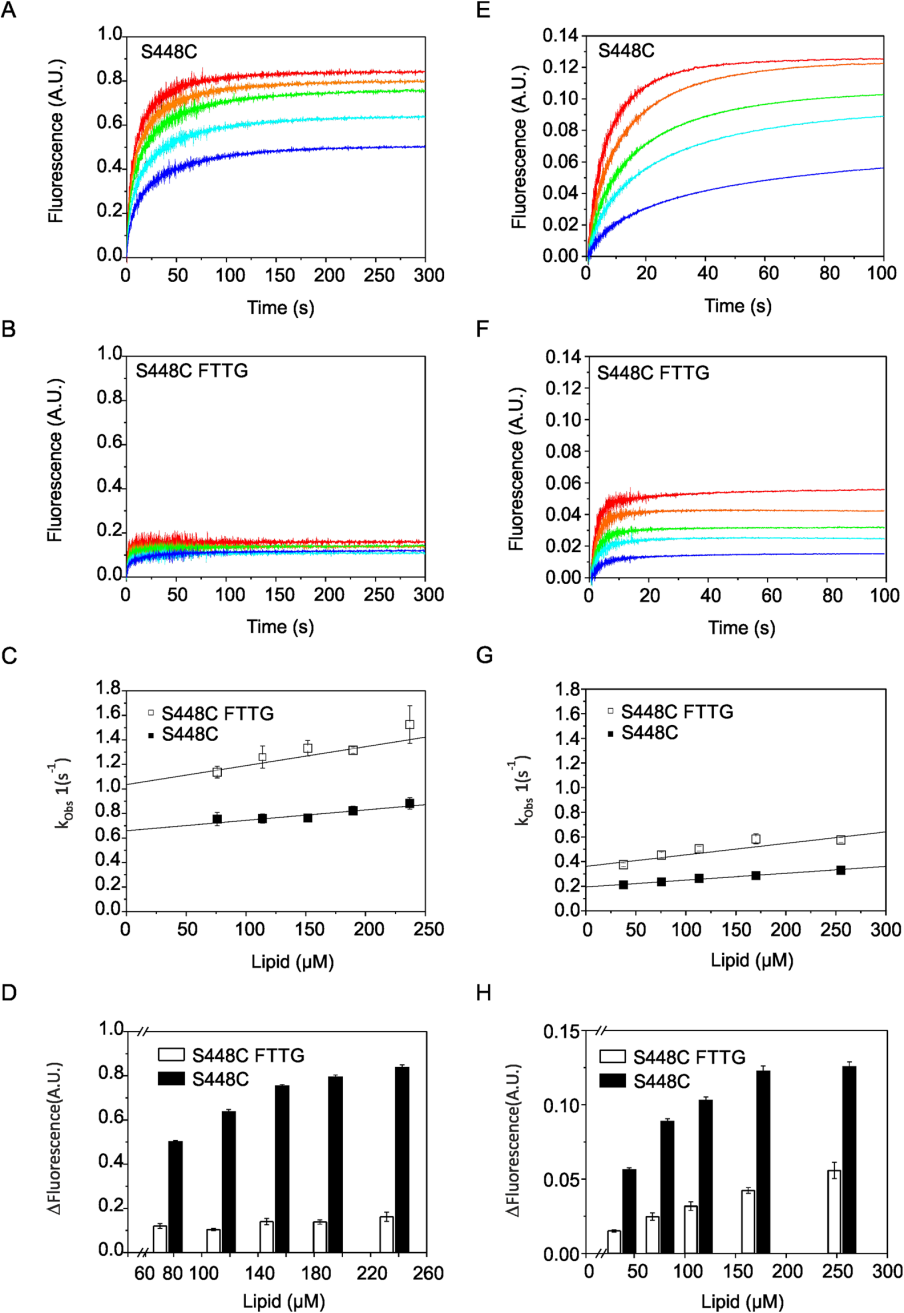
Stopped-flow measurements with S448C Atg18 and S448C FTTG Atg18. Time courses of Texas Red (**A** and **B**) or Rhodamine B (**E** and **F**) fluorescence emission measurements at different accessible lipid concentrations are shown for S448C Atg18 (**A** and **E**) and S448C FTTG Atg18 (**B** and **F**). A scale-up of the the traces in Fig. 2B and fitting results are shown in Supplementary Figure 11 and Supplementary Tables 3–7, respectively. (**C** and **G**) Graphs showing the dependency of *k*_obs1_ from the accessible lipid concentration. The y-intercept provides the dissociation rate constant *k*_off_, and the slope yields the association rate constant *k*_on_. Bars represent the standard errors of three to five technical repeats. The solid line shows a weighted linear fit (equation 2 in Methods). Amplitudes of the *k*_obs1_ used for data analysis are shown in Supplementary Figure 5. (**D** and **H**) Increases of Texas Red (**D**) or Rhodamine B (**H**) fluorescence emission are shown at different accessible lipid concentrations for S448C Atg18 and S448C FTTG Atg18.

The much lower FRET intensity of S448C FTTG indicates much weaker binding to liposomes in comparison with S448C Atg18 (Fig. 2A,B,D). The weak FRET signal observed for S448C FTTG makes accurate rate determination difficult and results in a higher standard error. However, no significant difference was observed in the association rate (*k*_on_) of both proteins, whereas the apparent dissociation rate (*k*_off,app_) was higher for the FTTG mutant (Table 2 and Fig. 2C) taking into account the experimental uncertainties (standard errors).

**Table 2:** Atg18 kinetic measurements.

	Alexa Fluor 488-Texas Red		Alexa Fluor 488-Rhodamine B	
	*k*_on_ (mM^−1^*s^−1^)	*k*_off,app_ (s^−1^)	*k*_on_ (mM^−1^*s^−1^)	*k*_off,app_ (s^−1^)
S448C	0.85 ± 0.22	0.66 ± 0.04	0.57 ± 0.38	0.19 ± 0.01
S448C FTTG	1.55 ± 0.39	1.03 ± 0.06	0.93 ± 0.20	0.36 ± 0.03

Rate constants for the kinetics of Atg18 binding calculated from data shown in Fig. 2C and F. The errors represent the standard error obtained from linear fitting.

We repeated the kinetic measurements with Lissamine Rhodamine B-PE instead of Texas Red-PE as the labelled lipid to further support our findings. Lissamine Rhodamine B-PE has a higher spectral overlap with Alexa Fluor 488 than Texas Red-PE, which allowed us to perform measurements at lower concentrations with a reliable signal/noise ratio. The final protein concentration was decreased from 0.25 µM to 0.125 µM in order to reduce aggregation during measurements (see below). Similar results were obtained under theses condition compared with the Texas Red-PE experiments, i.e. no significant difference in the association rates (*k*_on_) but a higher apparent dissociation rate (*k*_off,app_) was observed for the FTTG mutant in comparison with the S448C mutant (Table 2 and Fig. 2 E,F,G,H). These data demonstrate that initial membrane recruitment of PROPPINs is not mediated by PIP binding to the two PIP binding sites, suggesting non-specific electrostatic interactions with PIP molecules and negatively charged phospholipids mediate the membrane recruitment of PROPPINs.

### Analysis of membrane-protein distances with a fluorescence based liposome binding assay

In a previous study, we proposed a model for PROPPIN-membrane interactions, where the protein sits perpendicular on the membrane and binds through its two PIP binding sites on the rim of the β-propeller^13^. Here we set up two different fluorescence based liposome binding assays to probe the distances of selected residues towards the membrane in order to validate this model.

**Figure 3:**
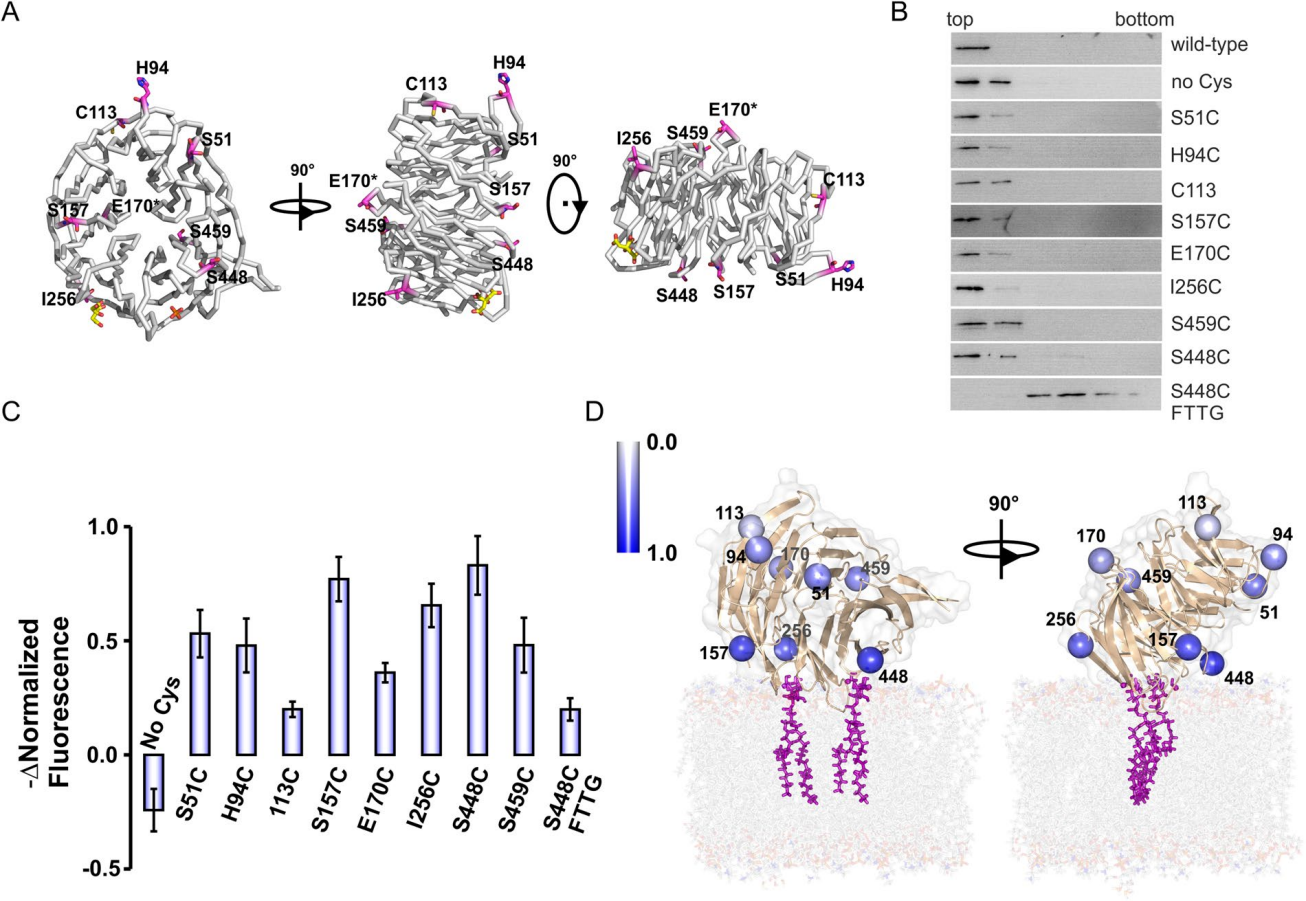
(**A**) Atg18 structure showing residues, which were mutated to cysteines for fluorescence labeling experiments. E170 is marked with an asterisk because its side chain is disordered in the structure and was modelled as an alanine. (**B**) Liposome flotation assays were performed with wild-type, cysteine-free and single cysteine Atg18 mutants using 100 nm LUVs composed of DOPC/DOPE/Texas Red-PE/PtdIns(3,5)P_2_ (76:21:2:1, molar ratio). The top two fractions of the gradient show protein bound to liposomes and bottom fractions contain unbound protein. The S448C FTTG mutant served as a negative control. (**C**) Observed changes of the normalized fluorescence at 520 nm for the Oregon Green labelled mutants after Texas Red liposome addition. (**D**) Model for Atg18 binding at the membrane. The docking was manually performed taking data from (**C**) into account. Residues are color coded according to the observed change in normalized fluorescence. Color bar is shown.

We prepared eight single-site cysteine mutants (Fig. 3A). Membrane binding of the mutants was confirmed through liposome flotation assays with LUVs containing 1% PtdIns(3,5)P_2_ (Fig. 3B, Supplementary Figure 7). In contrast, the S448C FTTG Atg18 mutant did not bind to liposomes, showing that protein-membrane binding is PIP specific. We measured the circular dichroism spectra of the mutants which confirmed their correct folding (Supplementary Figure 6).

First, cysteine residues were labelled with the environment sensitive dye 6-bromoacetyl-2-dimethylaminonaphthalene (BADAN). Fluorescence emission of BADAN is quenched in solution, however when the dye is in contact with a membrane its emission increases. BADAN-labelled proteins were incubated with LUVs containing 1% PtdIns(3,5)P_2_ for 10 min before acquisition of the fluorescence spectra. Proteins were mixed with the same buffer volume to obtain the fluorescence spectra in solution (Supplementary Figure 8). A large increase in fluorescence intensity upon addition of liposomes was observed for the S448C and S459C mutants. Both residues are close to PIP binding site 2. The I256C mutant in proximity of PIP binding site 1 showed no significant increase in fluorescence emission. One possible explanation is that this residue might not be inserted into the membrane and might instead be located at the membrane-aqueous interface. Of notice, the usage of the BADAN dye has several disadvantages, which includes a high unspecific labelling and different fluorescence intensities due to different local protein enviroments. To overcome these potential problems, we used a second complementary approach and performed FRET measurements with Oregon Green labelled protein and Texas Red labelled vesicles. The Oregon Green fluorescence in the presence of liposomes was monitored (Supplementary Figure 9). When the Oregon Green dye gets in proximity of the Texas Red labelled vesicles its fluorescence decreases. We observed the highest decrease in donor fluorescence for S448C, which is consistent with the BADAN measurements and also for I256C and S157C mutants, suggesting that these residues are closer to the membrane (Fig. 3C). Unexpectedly, a significant decrease was also observed for S51C and H94C mutants, suggesting that these residues are also in proximity of the membrane. Based on these FRET measurements we propose a manually docked model, where Atg18 binds the membrane perpendicular or slightly tilted, in contrast to an orientation where the β-propeller would lay planar on the membrane (Fig. 3D).

### Atg18 protein and liposome interactions analyzed by chemical cross-linking and mass spectrometry

To further characterize membrane binding of Atg18 we designed a chemical cross-linking strategy employing Bis(sulfosuccinimidyl)suberate (BS3). Samples were digested with trypsin and cross-linked di-peptides were then enriched by size-exclusion chromatography. We used a high-sensitivity, high-resolution mass spectrometer (Orbitrap Fusion Tribrid Mass Spectrometer) for liquid chromatography-coupled tandem mass spectrometry (LC-MS/MS) (see Methods for details). Potential cross-links were identified by database searching using the pLink software^26^.

**Figure 4:**
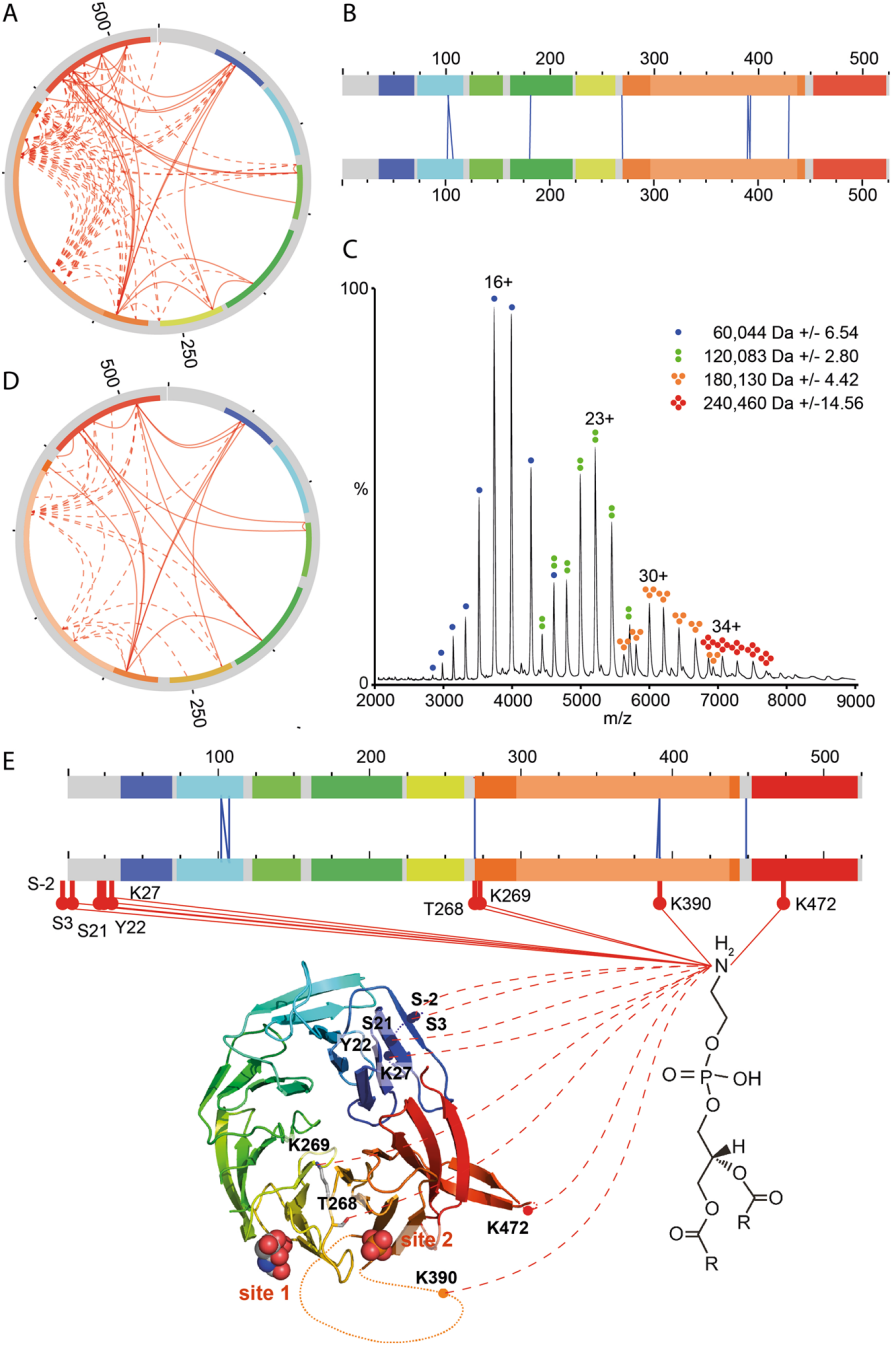
(**A**) Intramolecular cross-links of Atg18 are shown with red lines. Cross-links that are not included in the crystal structure are indicated by dotted lines. The seven blades of the β-propeller are rainbow colored. Residues missing from the crystal structure are highlighted in grey and light orange (residues 297–437, blade 6). (**B**) Bar diagramme indicating intermolecular Atg18 interactions (blue Lines). (**C**) Native mass spectrum of Atg18 oligomers. Calculated masses of the oligomers are given. (**D**) Intramolecular cross-links identified in Atg18 in the presence of liposomes. (**E**) Blue lines indicate intermolecular cross-links between two Atg18 molecules in the presence of liposomes. Red lines show protein-PE cross-links. PE cross-linked residues are labelled.

We verified unique cross-links corresponding to 101 protein interactions by evaluation of the tandem mass spectra (Supplementary Table 1). The intramolecular Atg18 cross-links are mainly located in loop regions missing in the Atg18 crystal structure suggesting flexibility of these regions (Fig. 4A). Interestingly, eight of the verified cross-linked di-peptides contained the same or overlapping peptide sequences showing that they derived from intermolecular cross-linking of two Atg18 molecules (Fig. 4B).

To gain further insights into possible oligomerisation of Atg18 we studied its intact assemblies by native mass spectrometry^27^. For this we employed a Q-ToF mass spectrometer modified for transmission of high-mass complexes^28^. Indeed, mass spectra of Atg18 reveal oligomers up to tetramers (Fig. 4C). Of these, monomeric Atg18 is most abundant, however, dimer, trimer and tetramer were obtained at reasonable intensities (50%, 20% and 10%, respectively).

We then incubated a mixture of Atg18 and LUVs composed of DOPC/DOPE/PtdIns(3,5)P_2_ (75:23:2, molar ratio) with BS3 following the same protocol as described above (Fig. 4A). In order to identify protein-lipid adducts, we performed a dependent database search allowing for unknown protein modifications (see Methods for details). The theoretical mass of a lipid adduct was calculated as follows: DOPE accounts for 743.36 Da and the BS3-linker for 140.074 Da; subtraction of two protons, which are released from the lipid and peptide amine groups during covalent linkage yields a mass adduct of 881.42 Da for a DOPE cross-linked peptide. Indeed, we identified several peptides with a mass adduct of 881.40 Da employing the MaxQuant software^29^. We assessed these potential hits by their peptide and modification site probability scores and thus approved four peptide sequences and nine cross-linked sites (Supplementary Table 2). Interestingly, in addition to cross-linked lysine residues we also identified several cross-linked serine, theronine and tyrosine residues (Fig. 4B). Cross-linking of these amino acids with BS3 was previously reported for peptides and proteins^30^.

The observed lipid-binding sites T268 and K269 are in proximity of the FRRG motif (residues 261–264) (Fig. 4E). K390 is localized in the membrane insertion loop 6CD, which is missing in the crystal structure. K472 localized in the disordered loop 7AB, which is nearby PIP binding site 2. Five cross-linked residues are present in the N-terminal region of Atg18 (S-2, S3, S21, Y22 and K27). The first 31 residues of Atg18 are disordered in the crystal structure and their flexibility allows interactions with the liposome membrane. Indeed, a different cross-linking pattern was observed for the N-terminal region of Atg18 either in the presence (Fig. 4D) or absence of liposomes (Fig. 4A). Various interactions that are formed in the absence of liposomes were not identified in the presence of lipids. In addition cross-links in close proximity to protein-lipid interactions (see above) were identified suggesting that the flexible N-terminal part of Atg18 interacts with the membrane.

We also identified 49 protein interactions within Atg18 and seven interactions specific for Atg18-Atg18 oligomers in the Atg18-liposome cross-linking experiments, which were also observed for BS3 cross-linking of Atg18 alone (Supplementary Table 1). The seven lysine residues, which formed intermolecular cross-links are K102 on strand 2C, K107 in loop 2CD and the disordered residues K181 in loop 4BC and residues K390, K392 and K429 in loop 6CD and one lysine in the N-terminal His-tag. Both, the detected intramolecular cross-links and the observation that Atg18 oligomers up to tetramers were measured in native mass spectrometry measurements show that Atg18 oligomerizes.

### Analysis of Atg18-Atg18 interactions through FRET based stopped-flow measurements

We performed FRET based stopped-flow measurements to further characterize Atg18-oligomerization. The protein was labelled with FRET dye pairs for these experiments. This set-up allowed us to study Atg18-Atg18 interactions either free in solution, in the presence of Ins(1,3,5)P_3_, which is the PtdIns(3,5)P_2_ head group, or with liposomes. We selected the C113 mutant for labelling, because this cysteine is nearby residues K102 and K107, which formed intermolecular cross-links.

**Figure 5:**
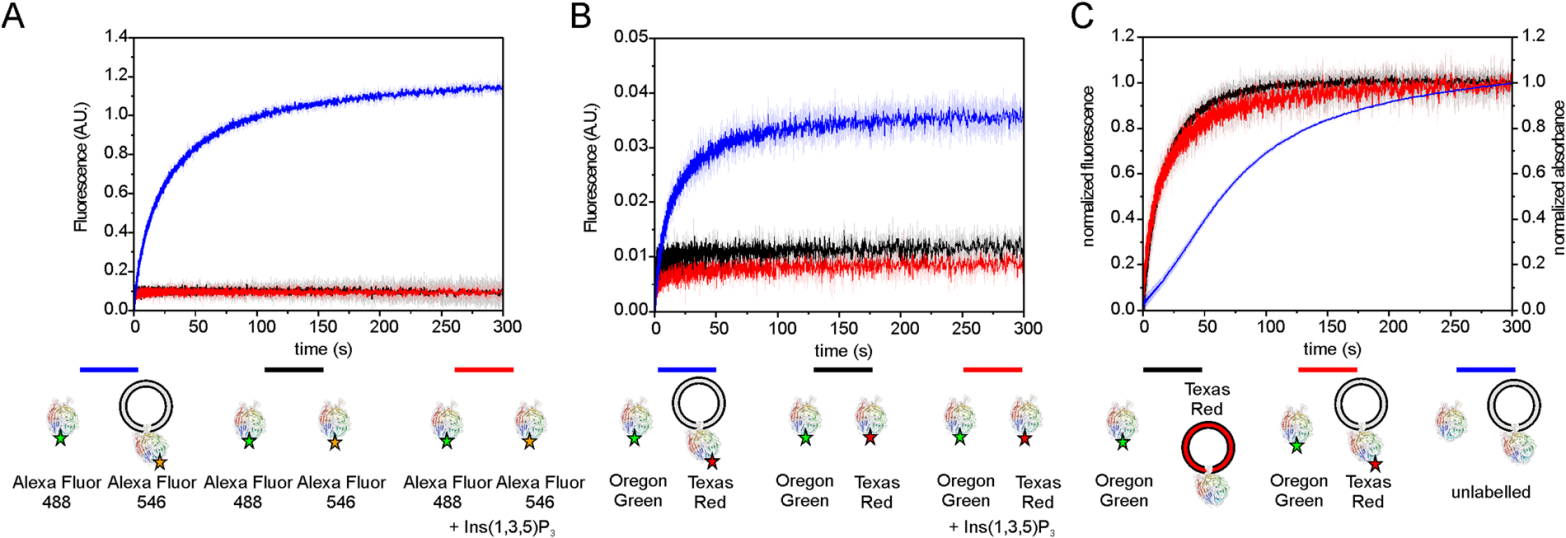
Characterization of Atg18-Atg18 interactions with FRET based stopped-flow measurements. (**A**) Graph shows time courses of rapid mixing of 0.5 µM Atg18 C113 (labelled with Alexa Fluor 488) with 0.5 µM Atg18 C113 (labelled with Alexa Fluor 546) either in the presence of unlabeled liposomes (1 mM total lipid), Ins(1,3,5)P_3_ (6 µM) or free in solution. (**B**) Experiments are similar to (**A**), but Oregon Green and Texas Red were used as donor and acceptor dyes instead. (**C**) Measurements with Texas Red labelled liposomes in comparison with measurements using labelled proteins for monitoring of the FRET signal. Unlabelled protein was mixed with unlabelled protein bound liposomes to monitor aggregation by measuring of the absorbance at 405 nm. Time course traces were normalized for comparison.

The Atg18 C113 mutant was either labelled with the FRET donor Alexa Fluor 488 or the acceptor dye Alexa Fluor 546. A very weak FRET signal was observed when measurements were done with the two labelled proteins in solution (Fig. 5A and Supplementary Figure 10B). However, measurements with C113-Alexa Fluor 546 labelled Atg18 bound to LUVs composed of DOPC/DOPE/Texas Red-PE/PtdIns(3,5)P_2_ (79:18:2:1, molar ratio) and C113-Alexa Fluor 488 labelled Atg18 free in solution yielded a strong FRET signal. In contrast, only a very weak FRET signal was observed for the two labelled proteins free in solution when Ins(1,3,5)P_3_ was present.

Stopped-flow measurements were repeated with another FRET dye pair. Here, the C113 mutant was labelled with either Oregon Green or Texas Red. Measurements were done as described above for the Alexa Fluor labelled proteins and gave consistent results (Fig. 5B). These data show that Atg18 oligomerizes when it is membrane bound. In contrast, the protein oligomerizes only to a small degree when free in solution and the Ins(1,3,5)P_3_ head group is not sufficient to mediate oligomerization. We cannot distinguish between dimerization and higher order oligomerization with these experiments.

In a control experiment aggregation of liposomes was monitored by injecting unlabelled proteins and liposomes and measuring the absorbance at 405 nm (Fig. 5C). Liposomes aggregate, however this process is slow in comparison to protein oligomerization. We also performed measurements with Texas Red labelled liposomes and Oregon Green labelled Atg18. The FRET signal evolves as rapidly as for the second measurement with Oregon labelled Atg18 and Texas Red labelled Atg18 bound to liposomes (Fig. 5C). These two experiments show that Atg18 oligomerization occurs very rapidly upon membrane binding of the protein.

## Discussion

We determined the 2.0 Å resolution crystal structure of *Pichia angusta* Atg18 with a citrate molecule bound in PIP binding site 1. The citrate interacts extensively with the protein (Fig. 1D). Earlier mutagenesis work showed that residues S241 and R248 which participate in citrate binding are essential for membrane binding of *S. cerevisiae* Atg18, i.e. the corresponding ScAtg18 residues are S264 and R271, respectively^13^. Bound citrates were also observed in PIP binding sites of other proteins such as the C-terminal PtdIns(3,4)P_2_ binding pleckstrin homology (PH) domain of human tandem PH-domain-containing protein 1 (TAPP1)^31^. Another example is the PtdIns3P binding FYVE domain from *Drosophila melanogaster* hepatocyte growth factor-regulated tyrosine kinase substrate (Hrs)^32^. The middle carboxylate group of the citrate bound Hrs structure (PDB: 1DVP) is near phosphate 3 in the Ins(1,3)P_2_ complexed human Hrs structure (PDB: 4AVX). In our Atg18 structures the middle carboxylate group is near the bound phosphate ion in site 1.

The phosphate ions in site 1 and 2 of the phosphate bound Atg18 structure are located at the same positions as the sulfate ions in *K. lactis* Hsv2 highlighting the strong conservation of the PIP binding sites^13,14^. While the overall structures of KlHsv2 and Atg18 are very similar, they vary in some of their loop regions and there are differences in the lengths of the strands in blade 7 (Supplementary Figure 1). There is an additional conserved region opposite of the PIP binding sites in Atg18, which includes loops 2AB and 2CD. Loop 2AB is involved in Atg2 binding as shown in mutagenesis studies with *S. cerevisiae* Atg18^15^. In contrast, the region comprising loops 1AB and 2AB is conserved in KlHsv2. These differences in surface conservation might be of functional importance, i.e. these sites might be involved in different protein-protein interations. Atg18 and Hsv2 differ in their autophagic functions. Atg18 is a core autophagy protein that is also required for various autophagic subtypes including the cytoplasm to vacuole targeting pathway and piecemeal microautophagy of the nucleus (PMN)^5,6,10^. In contrast, Hsv2 is only needed for PMN^8^.

We studied the influence of the FRRG motif on PROPPIN membrane binding kinetics by comparing the binding of Atg18 and its FTTG mutant to LUVs containing PtdIns(3,5)P_2_ with stopped-flow measurements. We used two different FRET dye pairs for these measurements (Fig. 2). We observed for both dye pairs that Atg18 and its FTTG mutant bind with similar association rates and that the FTTG mutant has a higher apparent dissociation rate. However, the absolute rate values should be interpreted with caution because Atg18 oligomerization might result in different equilibria which could affect with the final rate values obtained for the different protein concentrations. Both Atg18 oligomerization and aggregation affect the dissociation rate determination in our experimental set-up. If binding occurs in a two-step process, when omitting aggregation, then *k*_off,app_ in equation 2 (see Methods) can be defined as *k*_off,app_ = *k*_off_ + *k*_oli_ + *k*_dis_, where *k*_off_ is the dissociation rate of the binding step, *k*_oli_ is the rate of oligomerization and *k*_dis_ is the rate for oligomer disassembly. Here, the increase of *k*_off,app_ can be due to an increase of *k*_off_ or the two steps involved in oligomerization. When the first step of the process reaches equilibrium faster than the second one, as it is the case for diffusion-controlled process like membrane-binding processes then *k*_off,app_ ≈ *k*_off_ and *k*_obs1_ = *k*_on_[total accessible lipid] + *k*_off_. Furthermore, if *k*_oli_ + *k*_dis_ ≫ *k*_on_[total accessible lipid] or *k*_off_, then *k*_obs1_ is given by *k*_oli_ + *k*_dis_ and will not increase linearly with the concentration of the total accessible lipid^33^. However, the complexity of the binding process (binding and oligomerization) and the presence of early aggregation (Fig. 5C) adds uncertainty to determination of the real rates and must be interpreted with caution as stated above. Despite of this fact, our data are consistent with a mechanism for PROPPIN membrane binding kinetics, where initial membrane binding is driven by non-specific electrostatic interactions and once the PROPPIN is bound it is retained at the membrane by PIP binding of its two PIP binding sites.

Based on the crystal structure we designed mutants to further characterize Atg18-membrane binding by combining fluorescence based biophysical methods and mass spectrometry. We probed the distances of selected residues towards the membrane by firstly labelling cysteines with the environment-sensitive BADAN dye and secondly through FRET measurements with Oregon Green labelled Atg18 and Texas Red labelled vesicles. We observed an increase of BADAN fluorescence upon liposome binding for the labelled mutants S448C and S459C, which are near PIP binding site 2. Our FRET experiments showed the highest decrease in donor fluorescence and thus membrane binding for S448C, which is consistent with the BADAN measurements and additionally the S157C and I256C mutants, which are in proximity of both PIP binding sites. The observed differences between both independent measurements can be explained that BADAN is an environment-sensitive dye and Atg18 oligomerization could affect the local environment of BADAN similar to a vesicle membrane. Therefore, the results of the FRET measurements are more reliable, because here direct interactions between fluorophores are monitored, thus overcoming the potential problem of Atg18 oligomerization for the BADAN measurements. Our results are consistent with a perpendicular, or slightly tilted, binding of PROPPINs towards the membrane (Fig. 3D), confirming the earlier proposed model for PROPPIN-membrane binding^13–16^.

With a new approach we demonstrated that the amine-specific cross-linker BS3, which is commonly used for protein-protein cross-linking^34,35^ can also be used for protein-lipid cross-linking. We performed cross-linking experiments with Atg18 bound to LUVs containing PtdIns(3,5)P_2_ and detected DOPE-cross-linked lysine, serine, threonine and tyrosine peptides. Two residues, T268 and K269 are close to the FRRG motif. K390 is part of the membrane insertion loop 6CD.

**Figure 6:**
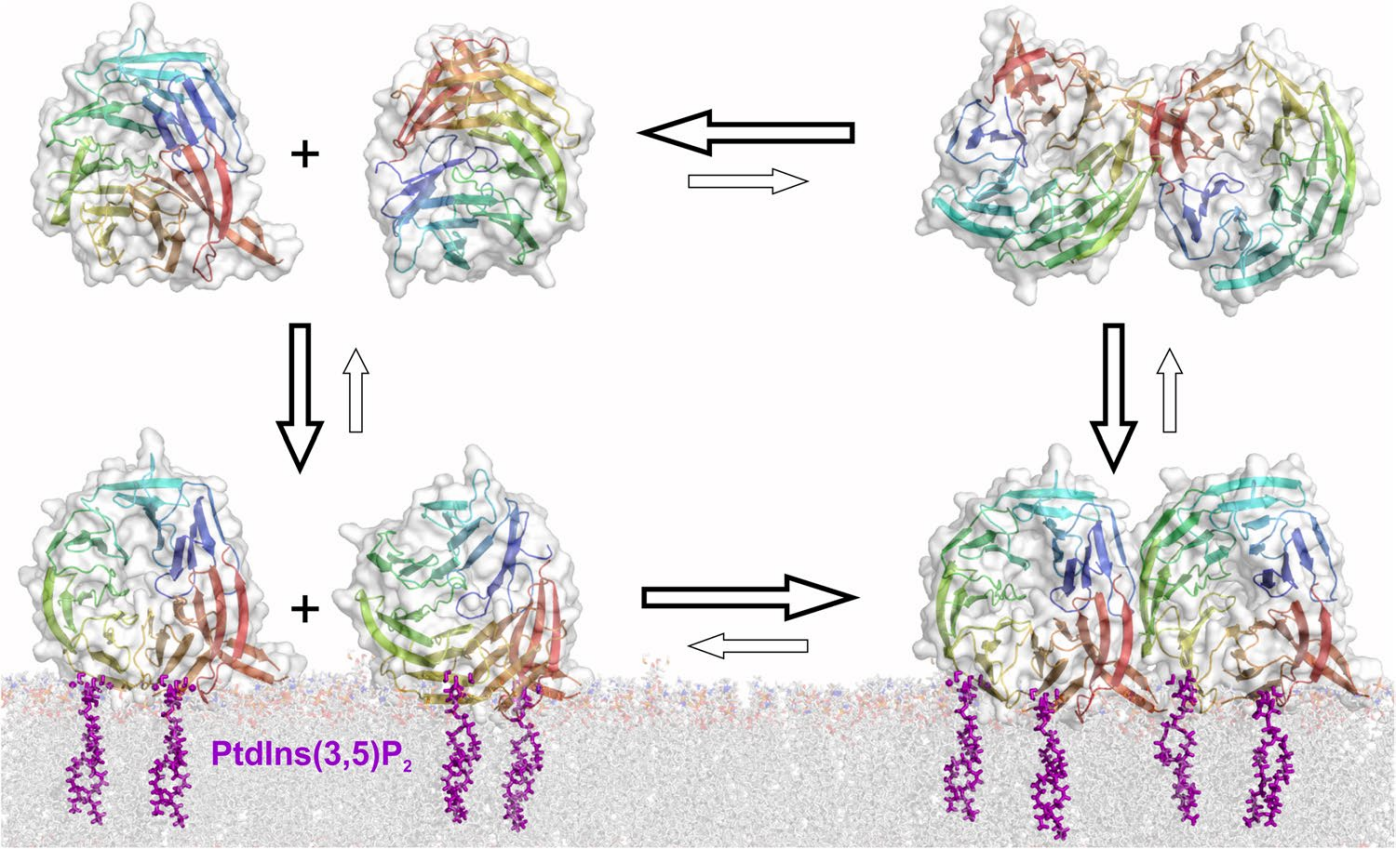
Model for Atg18 membrane binding. The protein oligomerizes upon membrane binding while it is mainly monomeric in solution. Higher order oligomers might be formed by membrane bound Atg18.

We also identified several intermolecular cross-linked peptides in our mass spectrometry analyses, which pointed towards an oligomerization of Atg18. We performed stopped-flow measurements to further investigate this finding. The stopped-flow measurements show that Atg18 oligomerizes upon membrane binding while the protein is mainly monomeric free in solution (Fig. 6). Atg18 oligomerization was also observed by cross-linking and native mass spectrometry of Atg18 in the absence of liposomes. The observed intensities of intact Atg18 oligomers in the native mass spectrometry measurements suggest an additive oligomerization mechanism and oligomerization is likely enhanced upon membrane binding as shown by stopped-flow measurements. A quantitative description of the two cross-linking experiments, in the presence and absence of LUVs, is not possible for two reasons. First, the cross-linking of Atg18 alone were performed with higher amounts of protein while the liposome binding experiments required lower protein concentrations, and second, we showed that BS3 cross-linker reacts with DOPE lipids in liposomes, which will also cause quenching of the cross-linking reaction (by two DOPE molecules cross-linking with each other) and a quantitative comparison is therefore not possible.

Oligomer formation occurs rapidly after membrane binding as identified by stopped-flow measurements and Atg18 bound liposomes aggregate later (Fig. 5C and Supplementary Figure 10A). In contrast, the protein oligomerizes only to a small extent in solution and the Ins(1,3,5)P_3_ head group is not sufficient to drive oligomerization (Fig. 5A,B and Supplementary Figure 10B).

The seven bladed β-propeller fold is rigid and therefore membrane binding will not induce large conformational changes of the protein. Membrane bound Atg18 molecules are arranged in the same perpendicular orientation through their two PIP binding sites on the rim of the β-propeller and membrane insertion of loop 6CD. Membrane binding leads to a large local concentration increase of the protein in comparison to an aqueous environment. Movement of the protein in the lipid bilayer is restricted to lateral diffusion. Taken together these three factors, i.e. protein concentration increase upon membrane binding, lateral diffusion and parallel orientation of membrane bound Atg18 molecules will promote protein oligomerization.

We cannot distinguish, whether Atg18 forms dimers or larger oligomers with the stopped-flow measurements. However, our native mass spectrometry results point towards an oligomerization beyond dimer formation because also trimers and tetramers were observed. In the cross-linking experiments two lysines, which are localized on opposite sites of the β-propeller, i.e. K102 on loop 2CD and K181 on loop 4BC formed intermolecular cross-links. K102 and K181 are localized in conserved regions of Atg18. These two observed intermolecular cross-links are compatible with a parallel side-to-side orientation of Atg18 oligomers, where neighboring molecules interact through their propeller rims (Fig. 6).

Membrane curvature has also an effect on PROPPIN binding. We previously showed that Hsv2 binds with an approx. 20 times higher affinity to SUVs containing PIPs than LUVs with the same composition^16^. Our results that membrane bound Atg18 oligomerizes and that membrane curvature promotes PROPPIN membrane binding explain the *in vivo* observation, that Atg18 localizes to the strongly bent edge of the isolation membrane in *S. cerevisiae* ^36^.

To conclude, our results presented here give further insights into the mechanism of Atg18 membrane binding. Atg18 is initially targeted to the membrane by non-specific PIP interactions and the FRRG motif is then important for retaining the protein at the membrane. Atg18 rapidly oligomerizes upon membrane binding and likely forms larger oligomers.

## Methods

### Protein expression and purification

A synthetic gene encoding *Pichia angusta* Atg18 (UniProt entry Q5QA94) cloned in the pET-28a vector was used for expression as described previously^16^. For the cysteine-free Atg18 mutant residues C45, C59, C65, C113 and C515 were mutated to alanines with the QuikChange Lightning Multi Site-Directed Mutagenesis Kit (Agilent Technologies). The S51C, H94C, C113, S157C, E170C, I256C, S459C and S448C single cysteine Atg18 mutants were prepared. Residues R262 and R263 were changed to threonines to generate the S448C FTTG single cysteine mutant. N-terminal His-tagged wild-type and mutant proteins were purified with a 1 mL HisTrap column (GE Healthcare) equilibrated with buffer A (20 mM imidazole, 0.3 M NaCl, 50 mM NaH_2_PO_4_ pH 7.5) and eluted with a gradient from 0 to 100% buffer B (0.4 M imidazole, 0.3 M NaCl, 50 mM NaH_2_PO_4_ pH 7.5). 2 mM 2-mercaptoethanol was added to the eluted protein. Concentrated protein samples were then loaded onto a HiLoad Superdex 200 16/60 column (GE Healthcare) using 0.3 M NaCl, 30 mM HEPES pH 7.5 as running buffer. Proteins were concentrated and frozen in liquid nitrogen. Selenomethionine-labelled wild-type Atg18 was expressed in selenomethionine containing minimal medium^37^ and purified with a HisTrap column using buffer A (20 mM imidazole, 500 mM NaCl, 50 mM HEPES pH 7.5) and buffer B (500 mM imidazole, 500 mM NaCl, 50 mM HEPES pH 7.5). In the second purification step, the protein was loaded onto a gel filtration column with a gel filtration buffer consisting of 300 mM NaCl, 30 mM HEPES pH 7.0.

### Crystallization and structure determination

Proteinase K was added in a 1:2000 (w/w) ratio to selenomethionine labelled Atg18 before setting-up the crystallization trials. Plate-like crystals grew with 2 µL 30 mg/ml protein and 2 µL 20% (w/V) PEG 8000, 0.2 M NaCl, 0.1 M sodium phosphate dibasic-citric acid pH 4.2 as precipitant (condition 31, Wizard 1 screen, Rigaku) in Linbro plates at 20 °C. Citrate-bound crystals were transferred into mother liquor containing 30% ethylene glycol. Phosphate-bound crystals were gradually transferred into a cryoprotectant consisting of 10% (w/V) xylitol, 10% (w/V) sucrose, 20% PEG 8000, 0.2 M NaCl, 0.4 M sodium dihydrogen phosphate pH 5.0. Crystals were flash-cooled in liquid nitrogen.

Diffraction data were collected at 100 K at the SLS beamline X10SA. Diffraction data were processed with the XDS software package^38^. The structure was determined by molecular replacement with *K. lactis* Hsv2 as a search model (PDB: 4AV8, residues 251–278 and 320–327 were deleted). Molecular replacement was done with Phaser-MR within PHENIX^39^. Two molecules were placed in the unit cell with a translation Z-score of 9.8. Automated model building was done with PHENIX. Manual rebuilding was done with COOT^40^ and the model was refined with REFMAC5^41^ and PHENIX^42^.

Figures were prepared with PyMOL^43^. Ligand-protein interactions were visualized with LigPlot+^44^. Protein sequences were aligned with ClustalOmega^45^ and analyzed with Consurf^46^. *P. angusta* Atg18, *S. cerevisiae* Atg18 (P43601), *Komagataella pastoris* Atg18 (Q8X1F5), *Scheffersomyces stipitis* Atg18 (A3GFE3), *Debaryomyces hansenii* Atg18 (Q6BIL4), *Meyerozyma guilliermondii* Atg18 (A5DHI9), *Candida albicans* Atg18 (Q5ABA6), *Candida glabrata* Atg18 (Q6FM63), *Aspergillus niger* Atg18 (A2RAG5), *Ustilago maydis* Atg18 (Q4P4N1), *Penicillium chrysogenum* Atg18 (A7KAM8), *Aspergillus terreus* Atg18 (Q0CW30), *Aspergillus clavatus* Atg18 (A1CBB8), *Magnaporthe oryzae* Atg18 (Q524W4), *Sclerotinia sclerotiorum* Atg18 (A7EW77), *Ashbya gossypii* Atg18 (Q75F47) and *Phaeosphaeria nodorum* Atg18 (Q0U2J8) were included in this analysis.

### Preparation of large unilamellar vesicles

Lipids were purchased from Avanti Polar Lipids and Texas Red-PE was obtained from Thermo Fisher Scientific. Lipid vesicles were generated by mixing of the different lipids chloroform stock solutions in the desired proportions. Lipids were dried in an oxygen-free nitrogen stream and the last traces of organic solvent were removed under vacuum in a vacuum desiccator for at least 3 h. Dried phospholipids were resuspended in buffer by vigorous vortexing. Large unilamellar vesicles with a diameter of around ~140 nm were prepared by extrusion of the rehydrated phospholipid suspensions with 21 strokes through a 0.1 µm polycarbonate membrane (Millipore Inc., Bedford, MA, USA) with a mini-extruder (Avanti Polar Lipids). Homogeneity of liposome preparations was confirmed by dynamic light scattering measurements with a DynaPro machine (Wyatt Technology).

### Liposome flotation assays

A 1 mg lipid mixture composed of DOPC/DOPE/PtdIns(3,5)P_2_/Texas Red-PE (76:21:1:2, molar ratio) was dried and then dissolved in 700 µL HP150 buffer composed of 150 mM KCl, 20 mM HEPES pH 7.4. LUVs were prepared with the mini-extruder using a 0.1 µm polycarbonate filter as described above. 5 µL 1 µM protein was mixed with 45 µL liposomes and incubated for 10 min at room temperature. 50 µL protein-liposome sample was then thoroughly mixed with 50 µL 80% (w/V) Nycodenz dissolved in HP150 and overlaid with 50 µL 30% (w/V) Nycodenz. 30 µL HP150 was added as top layer^47^. Samples were spun for 90 min at 55000 rpm (275000 × g) at 4 °C in a S55-S swinging bucket rotor in a Sorvall Discovery M150 SE analytical ultracentrifuge (Thermo Scientific). After centrifugation, 30 µL samples were taken from the gradient and analyzed by western blotting. Penta-His horseradish peroxidase conjugate antibody (Qiagen) was used in a 1:1500 dilution for detection.

### Protein labelling

Texas Red C2 Maleimide (Thermo Fisher Scientific), Oregon Green® 488 Maleimide (Thermo Fisher Scientific), Alexa Fluor 488 C5 Maleimide (Thermo Fisher Scientific), Alexa Fluor 546 C5 Maleimide (Thermo Fisher Scientific) and BADAN (Thermo Fisher Scientific) were used for specific labelling of the cysteine mutants. Proteins were incubated with a fivefold molar excess of the dye while gently shaking overnight at 4 °C. Unreacted dye was removed from the labeled protein with a PD-10 desalting column (GE Healthcare) using HP150 buffer with 0.1 mM TCEP.

### Fluorescence based liposome binding assay

LUVs composed of DOPC/DOPE/PtdIns(3,5)P_2_ (79:20:1, molar ratio) were prepared as described above. BADAN fluorescence emission was measured at 25 °C with a Fluorolog-3 spectrophotometer (Model FL322, HORIBA Jobin Yvon) in the range of 420 nm to 600 nm with an excitation wavelength of 380 nm and 5-nm slit width using 0.25 µM protein and ~500 µM total lipid.Three technical repeats were done for all proteins both in the presence and in the absence of vesicles. Standard deviations (SD) were calculated.

We prepared LUVs composed of DOPC/DOPE/Texas Red-PE/PtdIns(3,5)P_2_ (79:18:2:1, molar ratio) as described above for FRET measurements. Oregon Green fluorescence emission was measured similar to BADAN experiments in the range of 510 nm to 560 nm with an excitation wavelength of 490 nm.

### Stopped-flow experiments

Kinetic experiments were carried out with an Applied Photophysics SX.20 stopped-flow spectrophotometer at 25 °C in HP150 buffer with 0.1 mM TCEP as described previously^16^. Alexa Fluor 488 labelled Atg18 (0.5 µM or 0.25 µM) was mixed with equal volumes of increasing concentrations of Texas Red-labelled or Rhodamine B, LUVs (DOPC/DOPE/PtdIns(3,5)P_2_/labelled-PE (79:18:1:2, molar ratio)), under pseudo-first order conditions. The excitation wavelength was set on 495 nm and a 610 nm cut-off filter was used to collect the Texas Red emission for the different vesicle concentrations tested. The resulting time courses were fit to an exponential function:

1 $$F(t)={F}_{0}+\sum _{i=1}^{n}{A}_{obs(i)}\,\ast \,{e}^{-{k}_{obs(i)}\ast t}\,$$

where F(t) equals the observed fluorescence at time t, F_0_ is the final fluorescence, A_obs(i)_ represents the amplitude and *k*_obs(i)_ is the observed rate constant for the i th of n phases. Stopped-flow traces were evaluated using a three-exponential fitting, or bi-exponential at low protein concentration condition, yielding up to three *k*_obs_ or two *k*_obs_ respectively (Supplementary Figure 12). For data analysis, *k*_obs2_ and *k*_obs3_ were discarded due to aggregation during the time course of the measurements.

Observed rate constants were plotted as a function of accessible lipid concentration, assuming that only 60% of the total lipid concentration was accessible to the protein, and fitted with the equation:

2 $${k}_{{\rm{obs}}1}={k}_{{\rm{on}}}[{\rm{total}}\,{\rm{accessible}}\,{\rm{lipid}}]+{k}_{{\rm{off}},{\rm{app}}}$$

where *k*_on_ represents the association constant, and *k*_off,app_ the apparent dissociation rate constant.

For oligomerization experiments, we performed rapid mixing of acceptor fluorophore labelled Atg18 C113 (0.5 µM), in the presence of LUVs (1 mM total lipid), with (0.5 µM) of acceptor fluorophore labelled Atg18 C113 mutant (0.5 µM) in a 1:1 volume. In order to compare oligomerization with binding and aggregation, unlabelled Atg18 C113 in the presence of Texas red labelled LUVs (1 mM total lipid) were mixed with Oregon Green labelled Atg18 C113. The excitation wavelength was set on 495 nm and a 610 nm cut-off filter for all experiments. Similar conditions were used to monitor the aggregation with unlabelled protein and liposomes at 405 nm. Time courses were normalized for comparison.

### Statistical analyses

Statistical analyses were performed with unpaired *t* tests comparing test samples to wild-type in the same experiment, using the Graphpad QuickCalcs web (http://www.graphpad.com/quickcalcs/).

### Chemical cross-linking of Atg18 alone and with LUVs

Either approx. 14 µM or 1.4 µM Atg18 in 200 µL HP150 buffer alone or in the presence of LUVs composed of DOPC/DOPE/PtdIns(3,5)P_2_ (75:23:2, molar ratio) in 200 µL HP150 buffer were cross-linked with 2 µl 5 mM Bis[sulfosuccinimidyl] suberate (BS3) for 1 hr at 25 °C and 300 rpm. After cross-linking, the protein was precipitated with ethanol.

### Sample preparation for MS

The pellet from ethanol precipitation was dried in a vacuum centrifuge and dissolved in 1% (m/v) RapiGest SF Surfactant (Waters) in 25 mM ammonium bicarbonate. Atg18 was reduced with 10 µl 50 mM dithiothreitol for 1 hr at 37 °C and 300 rpm. Reduced cysteine residues were alkylated with 10 µl 100 mM iodoacetamide for 1 hr at 37 °C and 300 rpm. RapiGest was diluted with 25 mM ammonium bicarbonate to 0.1% (m/v) final concentration and Atg18 was digested with trypsin at a 1:20 enzyme: protein concentration ratio overnight at 37 °C. RapiGest was decomposed by addition of 20 µl 5% (v/v) trifluoric acid and incubation at 37 °C and 300 rpm for 2 hrs. RapiGest was removed by centrifugation at 13,000 rpm for 30 mins and the peptides were dried in a vacuum centrifuge.

Peptides were re-dissolved in 30% (v/v) acetonitrile, 0.1% (v/v) trifluoric acid. Linear peptides and cross-linked di-peptides were separated by size exclusion chromatography on a Superdex peptide column 10/300 GL (GE healthcare) at a flow rate of 50 µl/min. Fractions containing mostly cross-linked di-peptides were dried in a vacuum centrifuge and re-dissolved in 2% (v/v) acetonitrile, 0.05% (v/v) trifluoric acid.

### LC-MS/MS and database search to identify cross-linked peptides

Dissolved peptides were separated by nano-flow reversed-phase liquid chromatography (Dionex UltiMate 3000 RSLC, Thermo Scientific; mobile phase A: 0.1% (v/v) formic acid; mobile phase B: 80% (v/v) acetonitrile, 0.08% (v/v) formic acid). Peptides were loaded onto a trap column (Reprosil C18, 100 μm I.D., particle size 5 μm; Dr. Maisch GmbH, prepared in-house) and separated with a flow rate of 300 nL/min on an analytical C18 capillary column (Reprosil C18, 75 μm I.D., particle size 1.9 μm, 27–28 cm; Dr. Maisch GmbH, prepared in-house), with a gradient of 5–90% (v/v) mobile phase B over 75 min. Separated peptides were directly eluted into an Orbitrap Fusion Tribrid Mass Spectrometer (Thermo scientific).

Typical mass spectrometric conditions were: spray voltage of 2.5 kV; capillary temperature of 275 °C; normalized collision energy of 30% at an activation of q = 0.25 and an activation time of 30 ms. The Orbitrap Fusion Tribrid Mass Spectrometer was operated in data-dependent mode. Survey full scan MS spectra were acquired in the orbitrap (m/z 500–1700) with a resolution of 120,000 and an automatic gain control (AGC) target at 500,000. The top 20 most intense ions were selected for HCD MS/MS fragmentation in the orbitrap at an AGC target of 30,000 and with a first m/z of 110. Previously selected ions within previous 30 s were dynamically excluded for 20 s. Ions with charge states 3–8 were selected. Singly and doubly charged ions as well as ions with unrecognized charge state were excluded in order to increase the selection of highly-charged cross-linked peptides. Internal calibration of the orbitrap was performed using the lock mass option (lock mass: m/z 445.120025)^48^.

Raw data files were converted into mgf format using pXtract software tool (http://pfind.ict.ac.cn/software.html). Potential cross-links were then identified by database searching against a reduced database containing Atg18 using pLink search engine^26^. Search parameters were: fragmentation, HCD; enzyme, Trypsin; variable modifications, oxidation (methionine) and carbamidomethylation (cysteine); cross-linker, BS3. Spectra of potential cross-linked di-peptides were verified manually. Cross-links were visualised using the XV is software tool^49^.

### Mass spectrometry of intact Atg18 oligomers

The buffer of purified Atg18 was exchanged to 200 mM ammonium acetate using Micro Bio-Spin 6 gel filtration columns (Bio Rad). Intact Atg18 oligomers were then analyzed on a Q-ToF Ultima mass spectrometer modified for transmission of high mass complexes^28^ using gold-coated capillaries prepared in-house^50^. Typical mass spectrometric condition were: Capillary voltage, 1.7 kV; Cone voltage, 70 V; RF lens voltage, 180 V; collision energy, 25 V. Mass spectra were processed and analyzed using MassLynx 4.0 software.

### CD spectroscopy

CD measurements were performed using a Chirascan spectrometer (Applied Photophysics) for 0.2–0.3 mg/ml protein (monitored using Pierce™ 660 nm Protein Assay, Thermo Fisher Scientific) in 0.15 M NaF, 20 mM NaH_2_PO_4_ pH 7.5 (in a 0.1 cm Hellma quartz cuvette) between 190 and 260 nm at 25 °C (using 1 nm step size and 0.5 nm band width). Samples were centrifuged at 14000 rpm before measurements to remove aggregated and precipitated protein.

### Data availability

Coordinates and structure factors have been deposited in the Protein Data Bank with accession codes: 5LTD and 5LTG. Other data ara available from the corresponding authors upon reasonable request.

## Electronic supplementary material

Supplementary Information
