# Supplementary material for: Structure based biophysical characterization of the PROPPIN Atg18 shows Atg18 oligomerization upon membrane binding
Source: Sci Rep. 2017 Oct 25;7:14008. doi: 10.1038/s41598-017-14337-5 (PMC5656675; doi:10.1038/s41598-017-14337-5)
Supplement: Supplementary file 1 — Supplementary Information [file 41598_2017_14337_MOESM1_ESM.pdf]

Structure based biophysical characterization of the PROPPIN Atg18 shows Atg18 oligomerization upon membrane binding

Andreea Scacioc[1], Carla Schmidt[2,3*], Tommy Hofmann[2], Henning Urlaub[3,4], Karin Kühnel[1,6*] and Ángel Pérez-Lara[5*]

[1] Research Group Autophagy, Max-Planck-Institute for Biophysical Chemistry, Am Faßberg 11, 37077 Göttingen, Germany

[2] Interdisciplinary research center HALOmem, Martin Luther University Halle-Wittenberg, Kurt-Mothes-Str. 3, 06120 Halle, Germany

[3] Bioanalytical Mass Spectrometry Group, Max-Planck-Institute for Biophysical Chemistry, Am Faßberg 11, 37077 Göttingen, Germany

[4] Bioanalytics Group, University Medical Center Göttingen, Robert-Koch-Strasse 40, 37075 Göttingen, Germany

[5] Department of Neurobiology, Max-Planck-Institute for Biophysical Chemistry, Am Faßberg 11, 37077 Göttingen, Germany.

[6] present address: Nature Communications, 4 Crinan Street, London N1 9XW, United Kingdom

*address correspondence to the three corresponding authors: carla.schmidt@biochemtech.uni-halle.de, kkuehne@mpibpc.mpg.de, francisco-angel.perez-lara@mpibpc.mpg.de

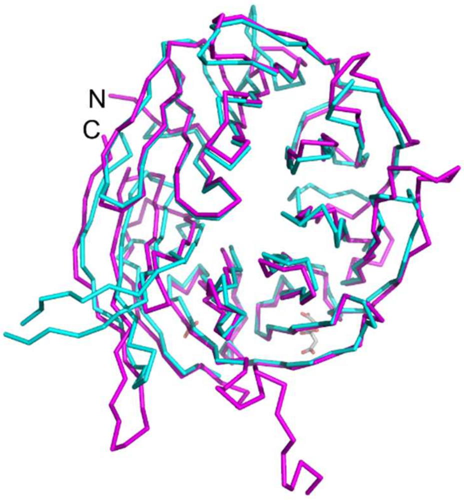

Supplementary Figure 1. Superimposition of the KlHsv2 (magenta) and PaAtg18 (cyan) structures. Citrate and phosphate ions bound in PIP binding sites 1 and 2 of PaAtg18 are shown.

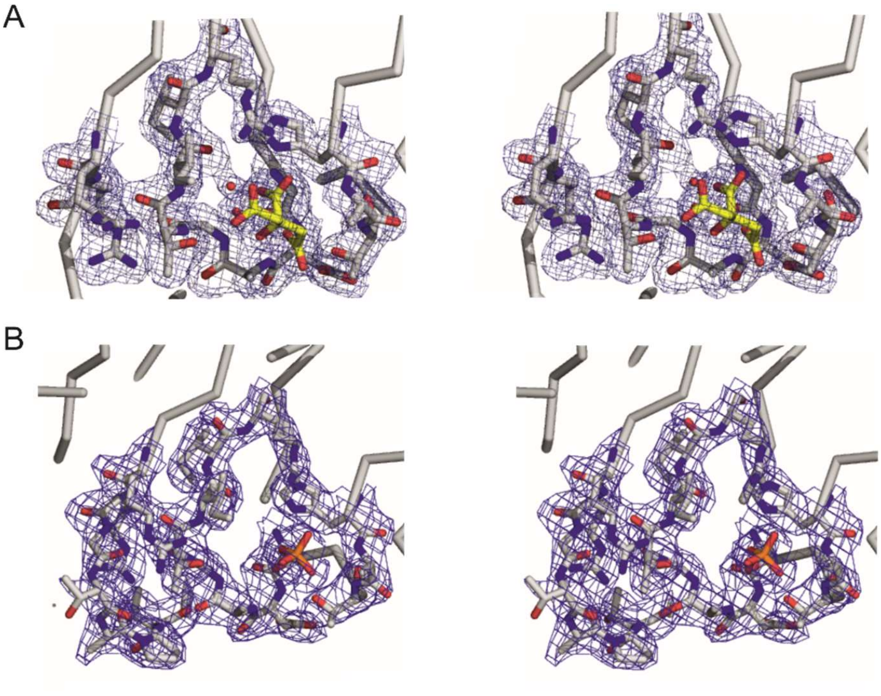

Supplementary Figure 2. Stereo view images showing (A) site 1 of the citrate bound structure and (B) site 1 of the phosphate bound structure with the overlaid 2mFo-DFc electron density maps contoured at σ=1.

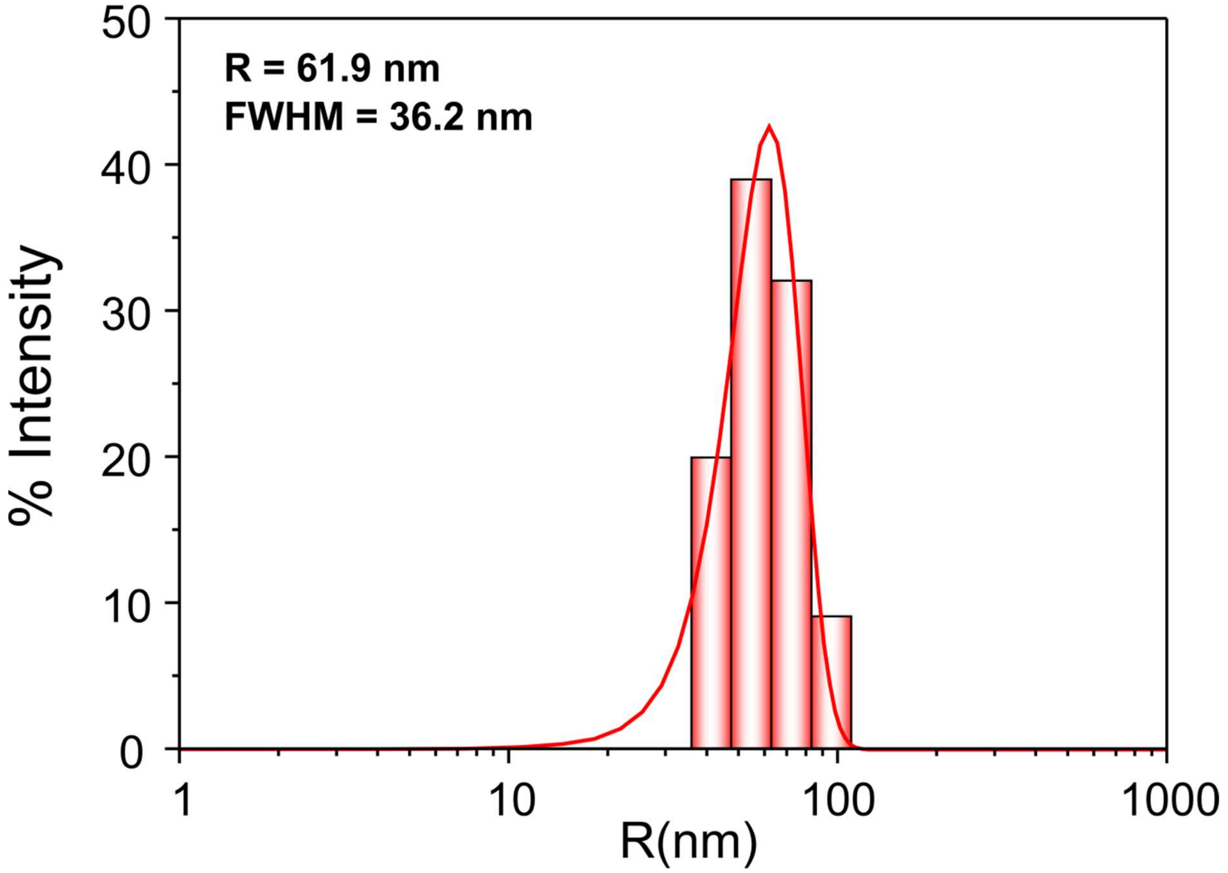

Supplementary Figure 3. Size distribution of LUVs composed of DOPC/DOPE/labelled lipid/PtdIns(3,5)P_2 (79:18:2:1, molar ratio) were determined with dynamic light scattering measurements using a DynaPro machine (Wyatt Technology). The mean and full width at half maximum (FWHM) of a Gaussian distribution fitting are shown.

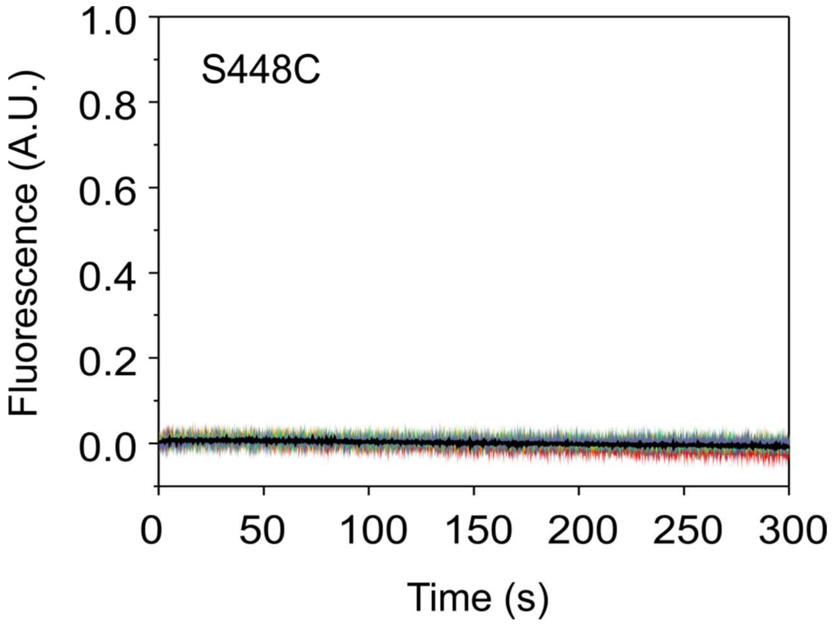

Supplementary Figure 4. The time course of Texas Red fluorescence emission for unlabelled S448C Atg18 in the presence of ~250 µM accessible lipid concentration shows that light scattering does not contribute to fluorescence signals measured in experiments presented in Figure 2.

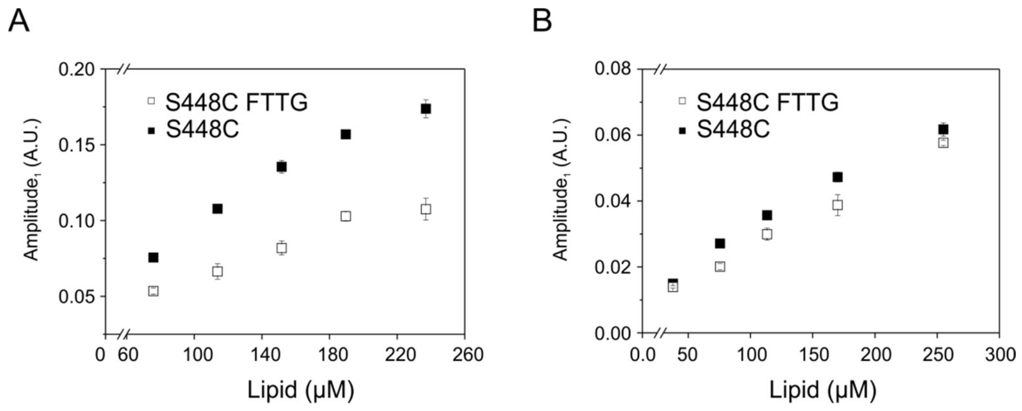

Supplementary Figure 5.Calculated amplitudes of the fitting for the fluorescence time courses from (A) Figures 2A,B and (B) Figures 2E,F at different accessible lipid concentrations.

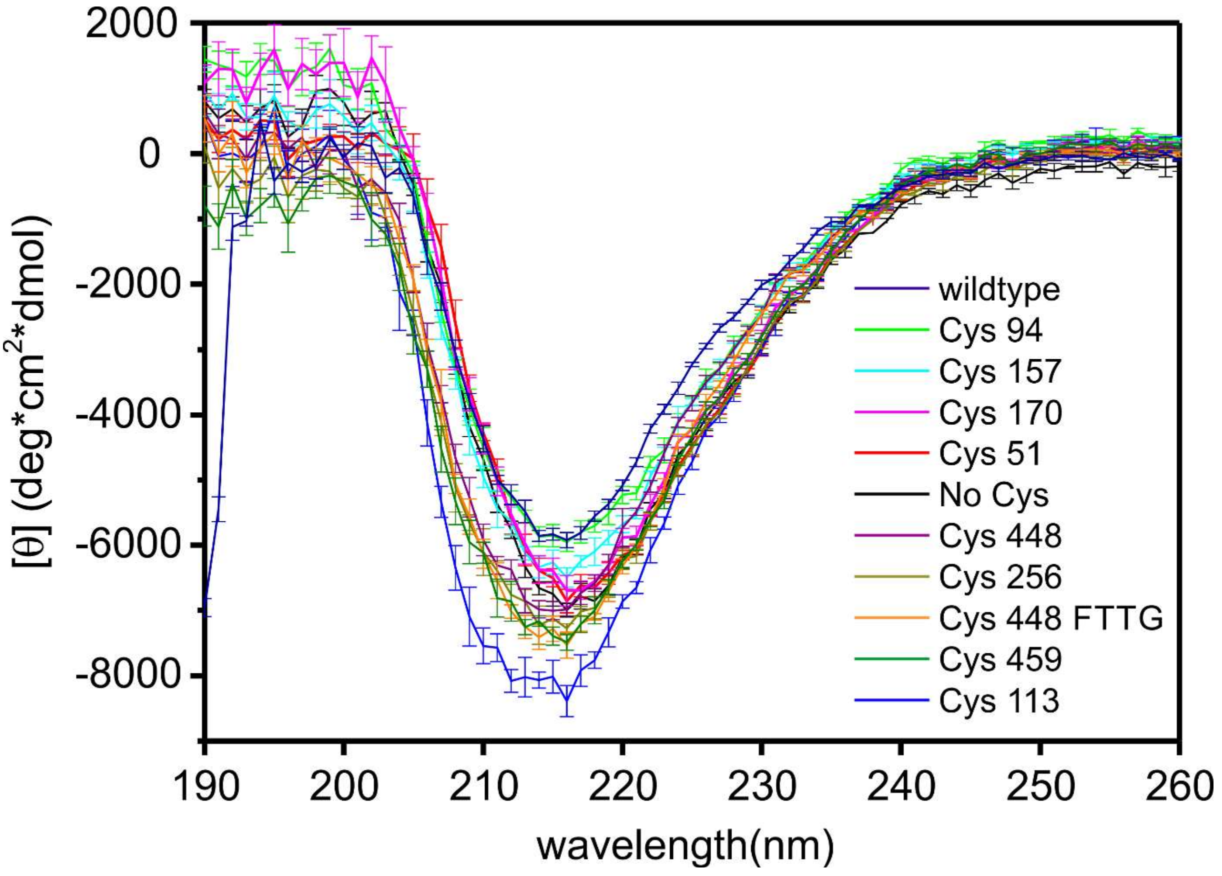

Supplementary Figure 6. CD spectra of Atg18 wild-type and Cys mutants. CD spectra were recorded for 0.2-0.3 mg/ml protein in 0.15 M NaF, 20 mM NaH_2PO_4 pH 7.5 at 25 °C.

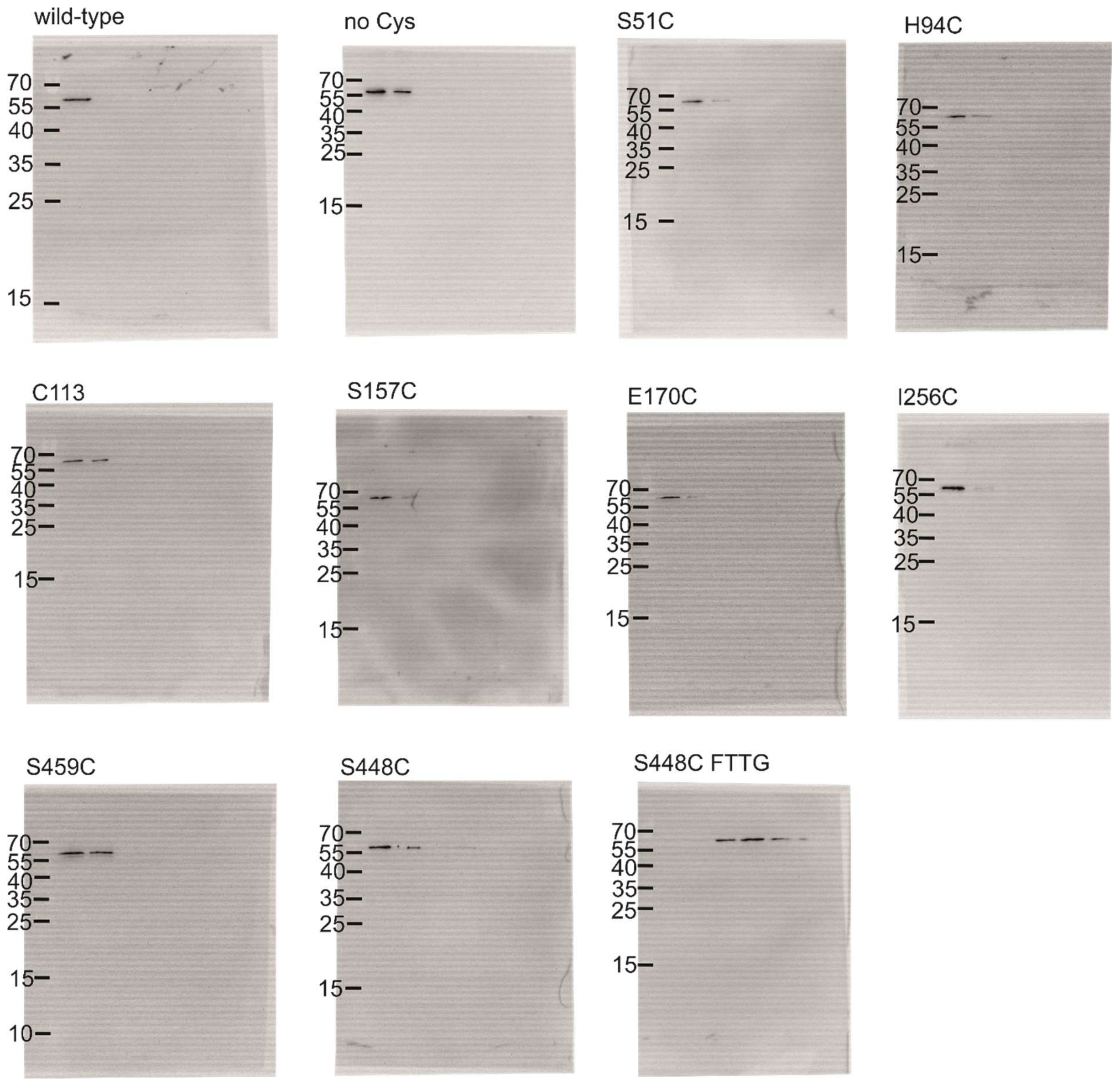

Supplementary Figure 7. Uncropped Blots of liposome flotation assays.

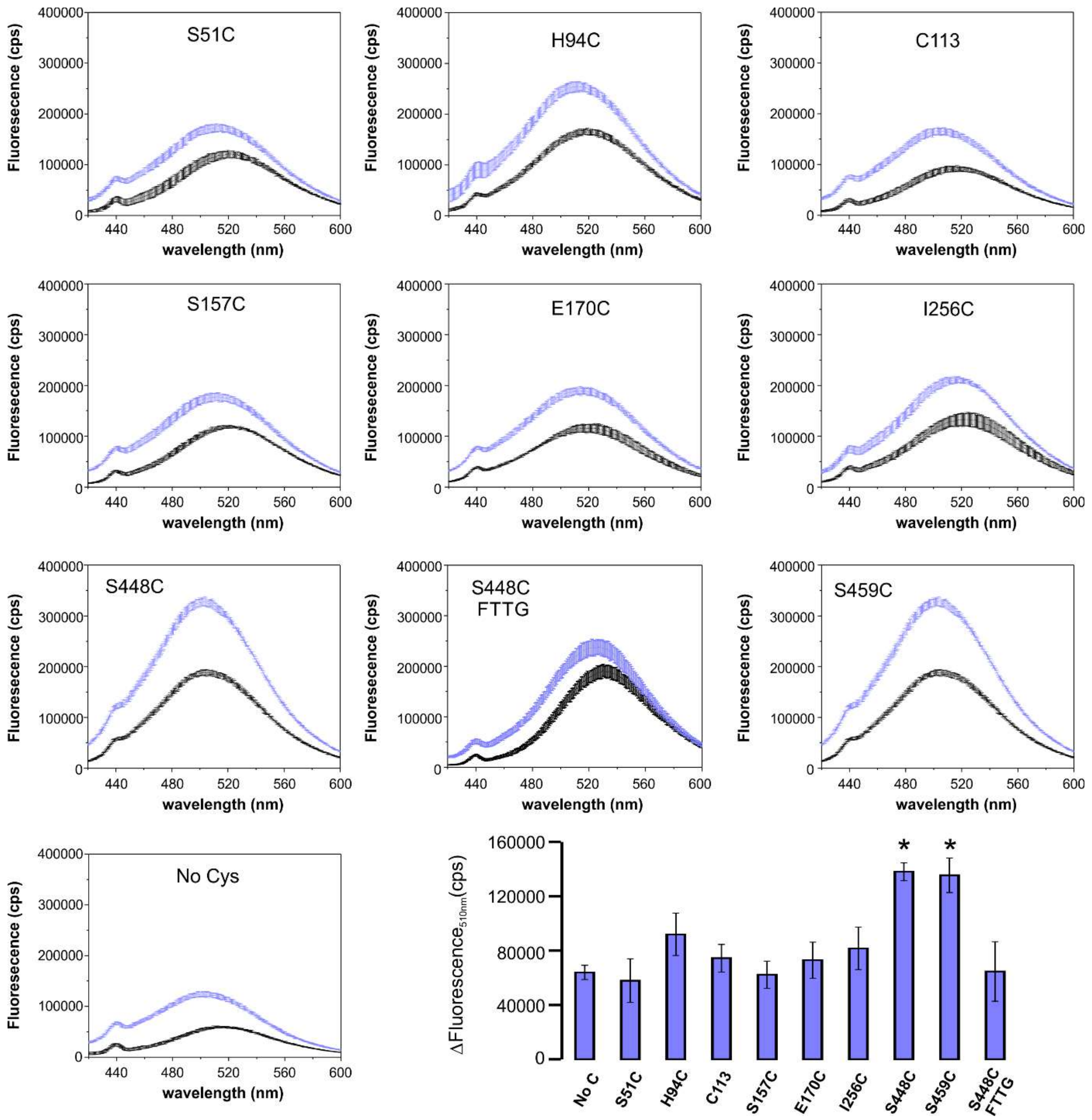

Supplementary Figure 8. Fluorescence spectra of BADAN-labelled Cys-mutants were recorded between 420 nm and 600 nm. Black traces show spectra acquired in the absence of liposomes and blue traces in the presence of liposomes. The increase of fluorescence at 510 nm in the presence of liposomes was quantified for all mutants. Error bars represents the S.D. for n =3 (*P < 0.01).

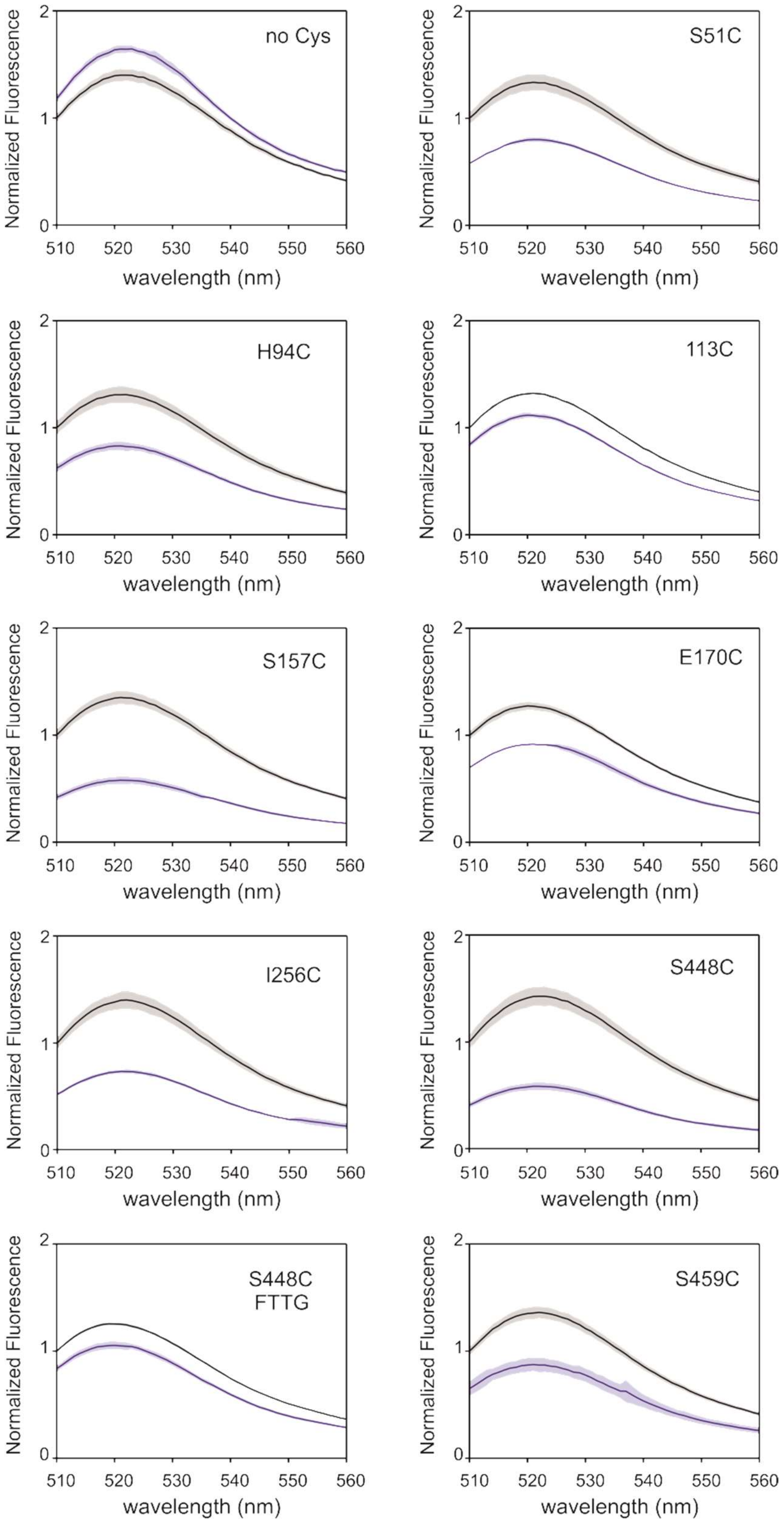

Supplementary Figure 9. Normalized fluorescence spectra of the Oregon Green-labelled Cys-mutants recorded between 510 nm and 560 nm. Black traces show spectra acquired in the absence of liposomes and blue traces measurements in the presence of Texas Red-labelled liposomes.

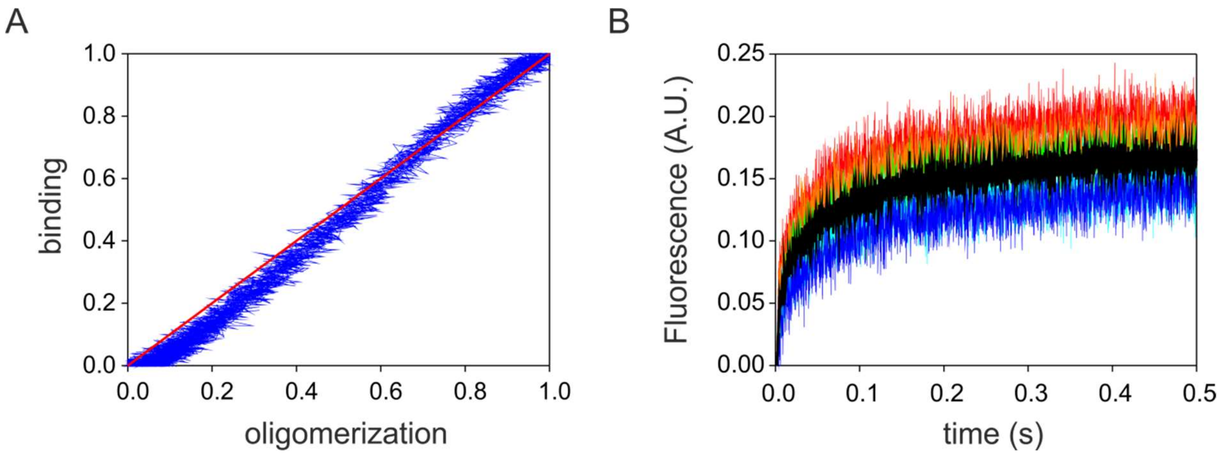

Supplementary Figure 10. (A) Plot of the normalized fluorescence of the binding versus oligomerization experiments from Figure 5C. (B) Atg18 oligomerization in the absence of vesicles. Experiment was performed as Figure 5A but using 1.5 μM final concentration of Atg18 in order to increase the signal/noise ratio. The traces correspond to the time courses emission of the different repeats (average trace in black).

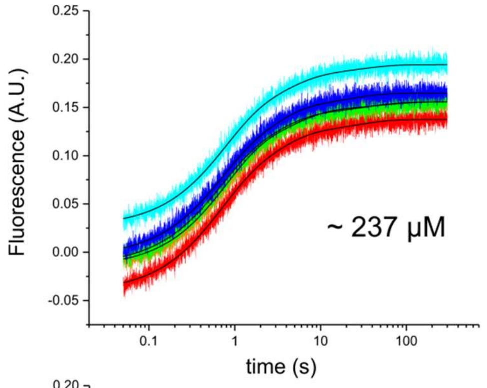
Fluorescence (A.U.)
0.25
0.20
0.15
0.10
0.05
0.00
-0.05
0.1
1
10
100
time (s)
~ 237 μM

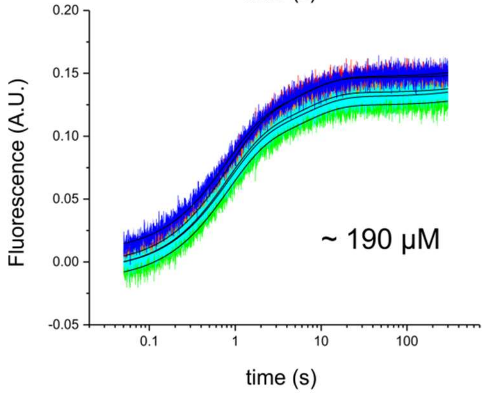
Fluorescence (A.U.)
0.20
0.15
0.10
0.05
0.00
-0.05
0.1
1
10
100
time (s)
~ 190 μM

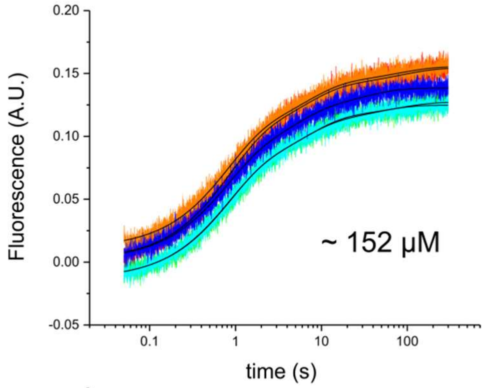
Fluorescence (A.U.)
0.20
0.15
0.10
0.05
0.00
-0.05
0.1
1
10
100
time (s)
~ 152 μM

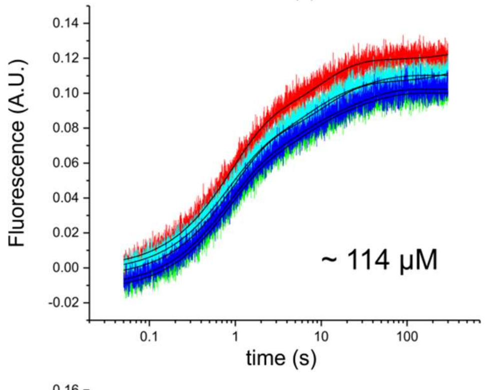
Fluorescence (A.U.)
0.14
0.12
0.10
0.08
0.06
0.04
0.02
0.00
-0.02
0.1
1
10
100
time (s)
~ 114 μM

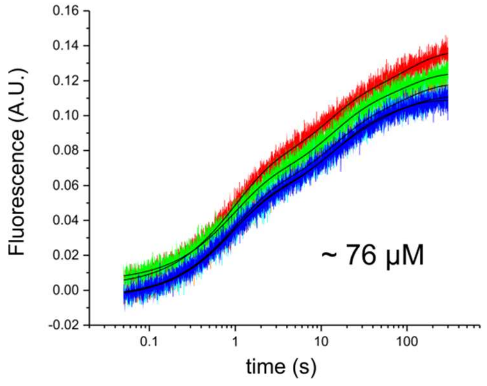
Fluorescence (A.U.)
0.16
0.14
0.12
0.10
0.08
0.06
0.04
0.02
0.00
-0.02
0.1
1
10
100
time (s)
~ 76 μM

Supplementary Figure 11. Time courses of the FTTG mutant stopped-flow replicates at different accessible lipid concentrations. Fittings to a three-exponential equation are shown with black lines. Low lipid concentrations show a pattern with three phases.

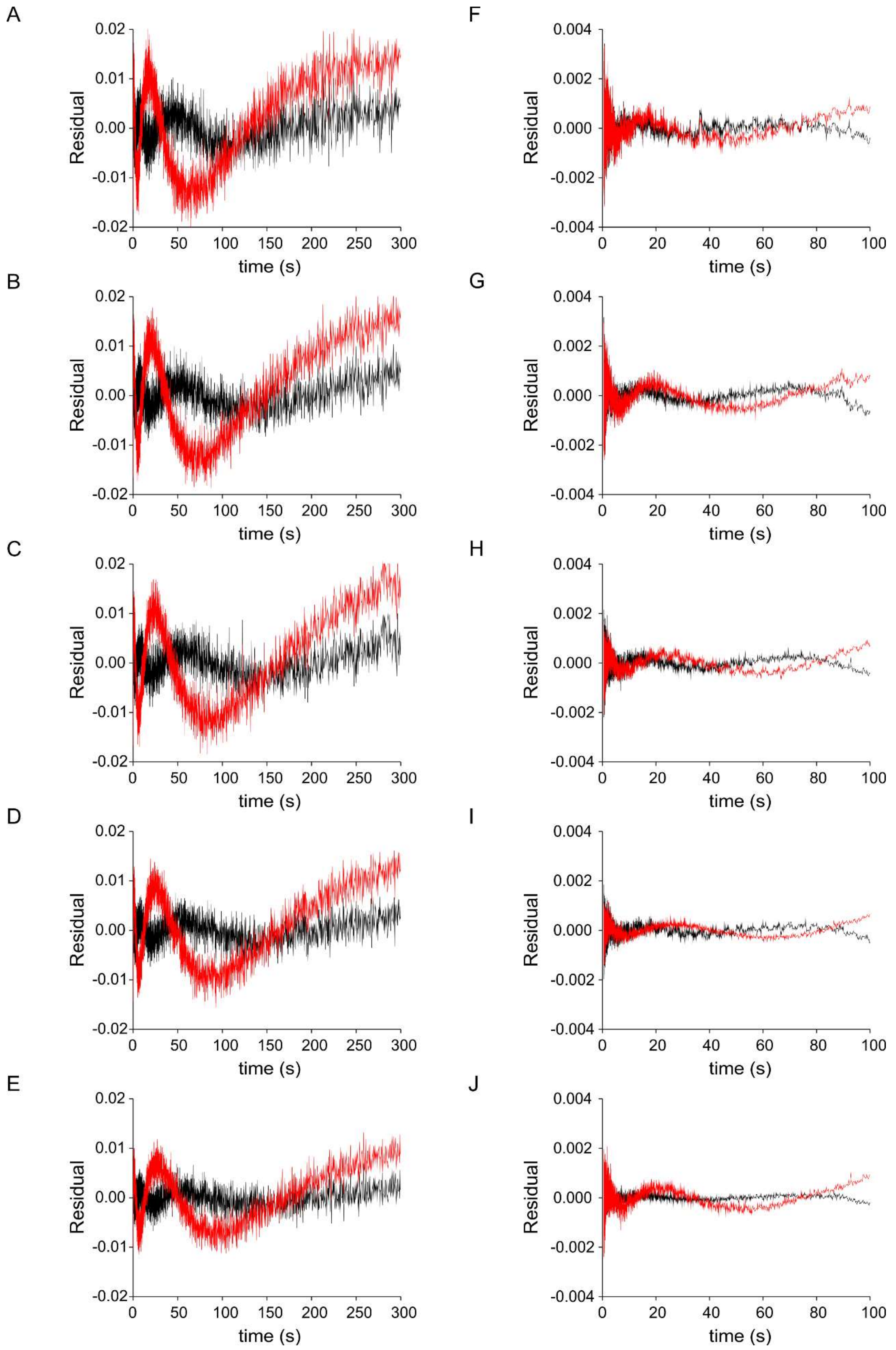

Supplementary Figure 12. Residual plots from two-exponential (red) or three-exponential (black) fits of time courses taken from data in Figure 2 panel A (A-E) and E (F-J). We observed a significant better fit to three-exponential equation for high protein concentration

(A-E) in contrast to low protein concentration, which presented no significant improvement (F-J).

Supplementary Table 1. Protein interactions obtained by chemical cross-linking with BS3 in Atg18 alone and in Atg18 in the presence of liposomes (LUVs). The cross-linked lysine residues are given. Intermolecular cross-links are marked grey.

Atg18			Atg18 + LUVs		
Residue 1	Residue 2	# spectra	Residue 1	Residue 2	# spectra
Lysine	Lysine		Lysine	Lysine	
27	30	144	27	30	24
			27	210	8
27	269	83	27	269	16
27	392	29	27	392	7
27	452	5			
27	472	14			
			27	488	8
30	210	8			
30	269	15	30	269	1
30	392	3	30	392	3
30	417	1			
30	401	6			
30	452	2			
			30	488	1
			102	102	1
102	390	6			
102	392	6			
102	488	3	102	488	2
102	107	3	102	107	1
107	107	13	107	107	6
107	316	2			
107	417	2			
107	444	2			
125	452	1			
181	181	2			
181	210	9			
181	243	7			
181	269	13	181	269	8
			181	392	2
			181	452	2
			181	488	1
210	269	5			
210	392	15	210	392	2
210	417	4			
			232	306	2
243	392	10			
243	269	8			
243	417	5			
257	306	28	257	306	1
257	392	5			

269	269	4	269	269	1
			269	306	6
269	390	3			
269	392	11			
269	401	61			
269	417	18			
269	444	13			
269	449	64	269	449	10
269	452	24	269	452	7
269	472	2			
306	392	16	306	392	11
306	401	5			
306	417	8			
306	421	2			
306	444	1			
306	429	3			
306	452	2			
			306	488	7
316	210	3	316	210	1
316	257	31	316	257	2
316	269	6	316	269	5
			316	306	7
316	390	3			
316	392	29	316	392	7
316	401	2			
316	417	7			
316	421	1			
316	429	17	316	429	3
316	452	2			
316	460	6			
316	472	1			
316	488	3	316	488	3
390	390	5			
390	392	13	390	392	2
390	401	12	390	401	6
390	417	10			
390	444	7			
			390	452	2
390	460	6			
			390	488	13
392	392	13	392	392	2
392	401	24			
392	417	7	392	417	2
392	421	6			
392	429	12			
392	444	9			
392	449	5			

392	452	3	392	452	4
392	460	8			
392	472	14			
392	488	10			
			392	489	2
401	444	1			
401	449	2			
401	452	1			
401	460	2			
401	472	2			
401	488	18			
417	429	12			
417	449	3			
417	452	1			
417	460	1			
417	488	15			
429	429	3			
			429	444	66
429	488	2			
444	452	9	444	452	3
444	472	5			
444	488	3			
			449	449	1
			452	417	1
452	472	1			
			452	488	2
			460	392	1
460	488	28	460	488	1
			472	392	3

Supplementary Table 2. PE-crosslinked peptides. The peptide sequences of cross-linked peptides are given. Cross-linked residues are highlighted in red. The residue number of the cross-linked residues, the dependent peptide (DP) scores and the probability of the cross-linked residue position are listed for each cross-linked peptide.

Peptide sequence	Residue no. in Atg18	DP Score	DP Positional Probability
GSHMASPNPLAFEAATAAHEVAASYVTEHKPR	3	253,98	0,9238179
GSHMASPNPLAFEAATAAHEVAASYVTEHKPR	21	262,63	0,9268981
GSHMASPNPLAFEAATAAHEVAASYVTEHKPR	3	306,58	0,9357136
IWDDLIPSVYLKDDANSITETSEDLVNKK	472	248,81	0,9431377
GSHMASPNPLAFEAATAAHEVAASYVTEHKPR	3	321	0,9483571
GSHMASPNPLAFEAATAAHEVAASYVTEHKPR	27	331,81	0,9560085
GTYPTKIYSLAFSPDNR	268/269	113,62	0,4823485
GSHMASPNPLAFEAATAAHEVAASYVTEHKPR	3	293,31	0,9957403
IWDDLIPSVYLKDDANSITETSEDLVNKK	472	244,63	0,938583
SSSSTGSFHSSESMTDKLKEPLVDNSR	390	183,65	0,7780715
GSHMASPNPLAFEAATAAHEVAASYVTEHKPR	-2	321	0,3313024
GSHMASPNPLAFEAATAAHEVAASYVTEHKPR	21	218,06	0,9251295
GSHMASPNPLAFEAATAAHEVAASYVTEHKPR	27	303,56	0,3310676
GSHMASPNPLAFEAATAAHEVAASYVTEHKPR	27	306,58	0,3308267
GSHMASPNPLAFEAATAAHEVAASYVTEHKPR	27	306,58	0,3308267
GSHMASPNPLAFEAATAAHEVAASYVTEHKPR	21/22	258,17	0,4853363
GSHMASPNPLAFEAATAAHEVAASYVTEHKPR	3	301,06	0,9489026
GSHMASPNPLAFEAATAAHEVAASYVTEHKPR	27	344,58	0,9571471
GSHMASPNPLAFEAATAAHEVAASYVTEHKPR	3	303,56	0,9416034
GSHMASPNPLAFEAATAAHEVAASYVTEHKPR	27	395,96	0,9781816
GSHMASPNPLAFEAATAAHEVAASYVTEHKPR	27	338,93	0,3315215

Supplementary Table 3. Results from FTTG mutants fitting to a three-exponential equation at 273 µM. Values used for data analysis are shown in red.

Model	stoppedflow3 (User)				
Equation	y = y0 - [A1*exp(-k1*x) + A2*exp(-k2*x) + A3*exp(-k3*x)] ;				
Reduced Chi-Sqr	2.97013E-5	2.93435E-5	3.02403E-5	3.02495E-5	2.93453E-5
Adj. R-Square	0.99071	0.99026	0.98926	0.98963	0.98986
		Value	Standard Error		
L	y0	0.13752	1.92219E-4		
	A1	0.06021	0.00274		
	A2	0.10143	0.00313		
	A3	0.01709	0.00101		
	k3	0.04132	0.00343		
	k1	0.39466	0.02462		
	k2	1.72891	0.04914		
M	y0	0.1559	2.98924E-4		
	A1	0.11544	0.00164		
	A2	0.04605	0.00144		
	A3	0.00989	7.14248E-4		
	k3	0.01953	0.00291		
	k1	1.40317	0.02417		
	k2	0.23361	0.01306		
N	y0	0.15563	2.19702E-4		
	A1	0.05281	0.00247		
	A2	0.10145	0.00272		
	A3	0.01493	7.67312E-4		
	k3	0.0309	0.00272		
	k1	0.36895	0.02286		
	k2	1.69832	0.04483		
O	y0	0.19418	2.01226E-4		
	A1	0.04792	0.00224		
	A2	0.01403	0.0013		
	A3	0.10568	0.00275		
	k3	1.47252	0.03677		
	k1	0.30863	0.02466		
	k2	0.03912	0.00455		
P	y0	0.16466	1.7757E-4		
	A1	0.06159	0.00323		
	A2	0.09008	0.00373		
	A3	0.01759	0.00116		
	k3	0.05054	0.00426		
	k1	0.43927	0.03019		
	k2	1.7928	0.06328		

Supplementary Table 4. Results from FTTG mutants fitting to a three-exponential equation at ~190 µM. Values used for data analysis are shown in red.

Model	stoppedflow3 (User)				
Equation	y = y0 - [A1*exp(-k1*x) + A2*exp(-k2*x) + A3*exp(-k3*x)] ;				
Reduced Chi-Sqr	2.45156E-5	2.5181E-5	2.42293E-5	2.43091E-5	2.37117E-5
Adj. R-Square	0.98985	0.9884	0.98854	0.98832	0.98867
		Value	Standard Error		
L	y0	0.90287	--		
	A1	0.10853	9.69331E-4		
	A2	0.75647	--		
	A3	0.0405	0.00102		
	k3	0.18656	0.00564		
	k1	1.30532	0.01749		
	k2	7.89692E-6	--		
M	y0	0.77025	158.21095		
	A1	0.03886	8.88852E-4		
	A2	0.63567	158.21067		
	A3	0.10238	9.18123E-4		
	k3	1.31785	0.01839		
	k1	0.1676	0.00655		
	k2	1.6404E-5	0.00409		
N	y0	0.78229	142.89208		
	A1	0.03772	8.40686E-4		
	A2	0.65779	142.8918		
	A3	0.10145	8.69686E-4		
	k3	1.30112	0.01765		
	k1	0.16106	0.00628		
	k2	1.69907E-5	0.0037		
O	y0	0.79113	117.14516		
	A1	0.1007	8.67987E-4		
	A2	0.03717	8.36763E-4		
	A3	0.65984	117.14488		
	k3	1.89754E-5	0.00338		
	k1	1.29453	0.01768		
	k2	0.1595	0.00634		
P	y0	0.78719	138.22387		
	A1	0.10108	8.46277E-4		
	A2	0.03841	8.16342E-4		
	A3	0.64	138.22358		
	k3	1.76581E-5	0.00382		
	k1	1.35864	0.01828		
	k2	0.16547	0.00627		

Supplementary Table 5. Results from FTTG mutants fitting to a three-exponential equation at ~152 μM. Values used for data analysis are shown in red.

Model	stoppedflow3 (User)				
Equation	y = y0 - [A1*exp(-k1*x) + A2*exp(-k2*x) + A3*exp(-k3*x)] ;				
Reduced Chi-Sqr	2.12165E-5	2.10804E-5	2.06547E-5	2.05697E-5	1.97466E-5
Adj. R-Square	0.99105	0.98999	0.98986	0.98972	0.98996
		Value	Standard Error		
L	y0	0.15434	4.27489E-4		
	A1	0.04264	8.57928E-4		
	A2	0.01586	5.18355E-4		
	A3	0.09429	9.54401E-4		
	k3	1.27022	0.01865		
	k1	0.1658	0.00719		
	k2	0.0124	0.00124		
M	y0	0.15545	3.91289E-4		
	A1	0.01482	5.58483E-4		
	A2	0.04244	8.10101E-4		
	A3	0.08697	8.82802E-4		
	k3	1.32907	0.02049		
	k1	0.0133	0.00139		
	k2	0.16325	0.00703		
N	y0	0.12726	3.78244E-4		
	A1	0.01365	5.80152E-4		
	A2	0.0836	8.95005E-4		
	A3	0.04309	8.18137E-4		
	k3	0.16031	0.00689		
	k1	0.01361	0.00154		
	k2	1.28711	0.02057		
O	y0	0.12472	2.1535E-4		
	A1	0.04085	0.00104		
	A2	0.081	0.00118		
	A3	0.01616	9.9243E-4		
	k3	0.02477	0.00219		
	k1	0.19608	0.01141		
	k2	1.34597	0.02558		
P	y0	0.13832	1.75955E-4		
	A1	0.07611	0.00152		
	A2	0.01967	0.00114		
	A3	0.04093	0.00125		
	k3	0.24272	0.01616		
	k1	1.42997	0.03278		
	k2	0.03367	0.00242		

Supplementary Table 6. Results from FTTG mutants fitting to a three-exponential equation at ~114 µM. Values used for data analysis are shown in red.

Model	stoppedflow3 (User)				
Equation	y = y0 - [A1*exp(-k1*x) + A2*exp(-k2*x) + A3*exp(-k3*x)] ;				
Reduced Chi-Sqr	1.67987E-5	1.6414E-5	1.6482E-5	1.59713E-5	1.62976E-5
Adj. R-Square	0.98947	0.9885	0.98803	0.98827	0.98808
		Value	Standard Error		
L	y0	0.83667	145.49248		
	A1	0.71802	145.49216		
	A2	0.08028	5.13144E-4		
	A3	0.03852	4.99675E-4		
	k3	0.10559	0.00316		
	k1	1.61971E-5	0.00329		
	k2	1.18013	0.01413		
M	y0	0.85032	65.09928		
	A1	0.03721	4.82334E-4		
	A2	0.74482	65.09889		
	A3	0.07392	4.71763E-4		
	k3	1.14727	0.0142		
	k1	0.09294	0.00299		
	k2	2.62429E-5	0.0023		
N	y0	0.10012	1.61234E-4		
	A1	0.0197	0.00166		
	A2	0.06501	0.00117		
	A3	0.02877	0.0013		
	k3	0.18948	0.01827		
	k1	0.03529	0.00293		
	k2	1.29101	0.02862		
O	y0	0.11029	1.5733E-4		
	A1	0.03084	0.00138		
	A2	0.01769	0.00177		
	A3	0.06428	0.00107		
	k3	1.33182	0.02843		
	k1	0.18478	0.01646		
	k2	0.0362	0.00342		
P	y0	0.10237	1.48711E-4		
	A1	0.02715	0.00131		
	A2	0.02375	0.0014		
	A3	0.06252	0.00162		
	k3	1.34774	0.03682		
	k1	0.24404	0.02628		
	k2	0.04026	0.00247		

Supplementary Table 7. Results from FTTG mutants fitting to a three-exponential equation at ~76 µM. Values used for data analysis are shown in red.

Model	stoppedflow3 (User)				
Equation	y = y0 - [A1*exp(-k1*x) + A2*exp(-k2*x) + A3*exp(-k3*x)] ;				
Reduced Chi-Sqr	1.22562E-5	1.25943E-5	1.23081E-5	1.24984E-5	1.25096E-5
Adj. R-Square	0.99301	0.99158	0.99139	0.99089	0.99068
		Value	Standard Error		
L	y0	0.13682	4.50739E-4		
	A1	0.03992	7.03216E-4		
	A2	0.03139	6.0876E-4		
	A3	0.06333	4.71166E-4		
	k3	1.11314	0.01481		
	k1	0.09667	0.00355		
	k2	0.01081	6.05743E-4		
M	y0	0.1183	4.3258E-4		
	A1	0.05595	4.30153E-4		
	A2	0.02693	9.00561E-4		
	A3	0.03944	9.8234E-4		
	k3	0.08255	0.00344		
	k1	1.08983	0.01579		
	k2	0.01168	8.54075E-4		
N	y0	0.12439	4.25334E-4		
	A1	0.04106	9.27825E-4		
	A2	0.02506	8.43716E-4		
	A3	0.05316	4.1558E-4		
	k3	1.13791	0.01707		
	k1	0.08454	0.00325		
	k2	0.01164	8.83658E-4		
O	y0	0.11109	4.04349E-4		
	A1	0.04094	0.0012		
	A2	0.0209	0.00112		
	A3	0.05385	3.9684E-4		
	k3	1.12847	0.0164		
	k1	0.07718	0.00327		
	k2	0.01244	0.00122		
P	y0	0.10876	2.23816E-4		
	A1	0.05101	4.97725E-4		
	A2	0.03469	0.00126		
	A3	0.02764	0.00138		
	k3	0.01931	0.00116		
	k1	1.21481	0.02095		
	k2	0.10386	0.00573		
